# Genetic variation and forensic efficiency of autosomal insertion/deletion polymorphisms in Chinese Bai ethnic group: phylogenetic analysis to other populations

**DOI:** 10.18632/oncotarget.17137

**Published:** 2017-04-17

**Authors:** Chun-Hua Yang, Cai-Yong Yin, Chun-Mei Shen, Yu-Xin Guo, Qian Dong, Jiang-Wei Yan, Hong-Dan Wang, Yu-Dang Zhang, Hao-Tian Meng, Rui Jin, Feng Chen, Bo-Feng Zhu

**Affiliations:** ^1^ Key Laboratory of Shaanxi Province for Craniofacial Precision Medicine Research, College of Stomatology, Xi'an Jiaotong University, Xi'an, Shaanxi, P. R. China; ^2^ Clinical Research Center of Shaanxi Province for Dental and Maxillofacial Diseases, College of Stomatology, Xi'an Jiaotong University, Xi'an, Shaanxi, P. R. China; ^3^ Department of Forensic Genetics, School of Forensic Medicine, Southern Medical University, Guangzhou, Guangdong, P. R. China; ^4^ Department of Forensic Medicine, Nanjing Medical University, Nanjing, Jiangsu, P. R. China; ^5^ Institute of Brain and Behavioral Sciences, College of Life Sciences, Shaanxi Normal University, Xi'an, Shaanxi, P. R. China; ^6^ Key Laboratory of Genome Sciences, Beijing Institute of Genomics, Chinese Academy of Sciences, Beijing, P. R. China; ^7^ Medical Genetic Institute of Henan Province, People's Hospital of Henan Province, Zhengzhou, Henan, P. R. China; ^8^ Institue of Forensic Sciences of Anhui Public Security Department, Hefei, Anhui, P. R. China; ^9^ Department of Radiology, The Second Affiliated Hospital of Xi'an Jiaotong University, Xi'an, Shaanxi, P. R. China

**Keywords:** Indel, Bai group, phylogenetic analysis, population genetics

## Abstract

Thirty insertion/deletion loci were utilized to study the genetic diversities of 125 bloodstain samples collected from Bai group in Yunnan Dali region, China. The observed heterozygosity and expected heterozygosity of the 30 loci ranged from 0.1520 to 0.5680, and 0.1927 to 0.4997, respectively. No deviations from Hardy-Weinberg equilibrium tests after Bonferroni correction were found at all 30 loci in Bai group. The cumulative probability of exclusion and combined discrimination power were 0.9859 and 0.9999999999887, respectively, which indicated the 30 loci could be used as complementary genetic markers for paternity testing and were qualified for personal identification in forensic cases. We found the studied Bai group had close relationships with Tibetan, Yi and Han groups from China by the population structure, principal component analysis, population differentiations, and phylogenetic reconstruction studies. Even so, for a better understanding of Bai ethnicity's genetic milieu, DNA genotyping at various genetic markers is necessary in future studies.

## INTRODUCTION

As a new system of diallelic genetic marker, insertion/deletion (Indel) polymorphism, also called DIP, has recently been used in forensic sciences and population genetic studies. Due to the simple construction, Indels can be amplified to small amplicons and even degraded DNA samples can be analyzed accurately. Also, Indels have low mutation rates [1, 2]. Additionally, the genotyping method of Indels is similar to that of STRs comprising polymerase chain reaction (PCR) and capillary electrophoresis (CE) and easily achieved in forensic biology laboratories. In our study, InDels are diallelic markers which show 2 different alleles (+ or -), the number of InDel alleles is much less than those of STRs (for instance, D5S818 locus, allele 7, 8, 9, 10, 11, 12, 13 and 14). As a result, the forensic parameters of InDels, like discrimination power (DP), are lower than those of STRs with the calculation formula, $\text{DP}=1-\sum_{\text{i}=1}^{\text{n}}{\text{P}}_{\text{i}}^{2}$ [3]. We could infer that the DP values are increased with the more alleles through integral theorem which was employed to make the explanation more comprehensive and objective. Therefore, Indels could be utilized as complement markers to improve the efficiency of existing genetic markers in personal identification and paternity testing. After Weber et al. reported human diallelic Indel polymorphisms for the first time [4], Indels have also drawn much attention in the biogeogarphic field 5–7]. For multiplex amplification of 30 Indel loci plus a sex-determining locus Amelogenin, Qiagen Investigator DIPplex kit (Qiagen, Hilden, Germany) which has recently been made commercially available was employed in our study [8].

According to the 6^th^ nationwide population census of the People's Republic of China, Bai, with 1,933,510 people, ranks the 15^th^ of the 56 ethnic groups (http://www.stats.gov.cn/tjsj/pcsj/rkpc/6rp/indexce.htm). Approximately 80% of Bai people live in concentrated communities distributed in the Dali Bai Autonomous Prefecture in Yunnan Province, southwest China. Archaeological findings indicated that the history of Bais could be traced back to the Neolithic Age. Bai's language originated from Sino-Tibetan language family. The language of Bai maintains lots of Han words due to the Bais' long-term cultural contact with Han, the largest Chinese ethnic group. Bai ethnic group became a member of China, preserving their own the science and culture, since the Western Han Dynasty (109 BC) [9].

In this research, we obtained population data and forensic parameters of 30 Indels in the Bai ethnic group, and then analyzed the genetic differentiation between the Bai ethnic group and other populations.

## RESULTS

### Linkage disequilibrium analysis

**Figure 1:**
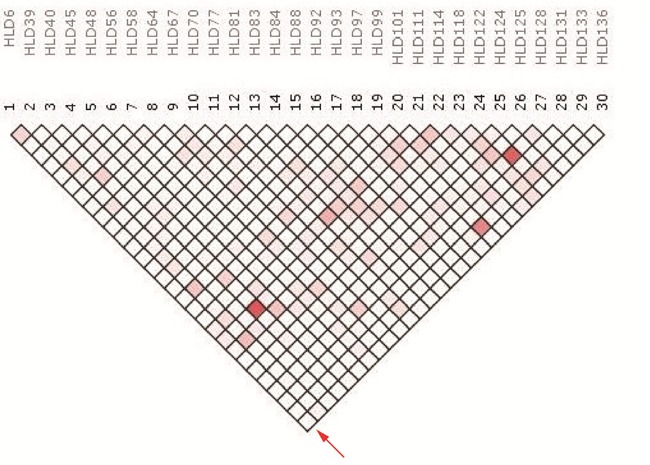
The LD analysis schema between the 30 Indel loci utilizing the SNPAnalyzer 2.0 program

The results of LD (linkage disequilibrium) were shown on the form of an inverted triangle (Figure 1). As shown by the red arrow, the pairwise LD between the locus HLD6 and the rest 29 loci were arranged in the left bottom (29 square grids). The others were performed so on. No red color area embraced with thick black curve was observed in the linkage disequilibrium graph.

### Forensic parameters

The allele frequencies and forensic statistical parameters of the 30 Indel loci in Chinese Bai ethnic group were exhibited in Table 1, and the detailed Indel genotypes were shown in Supplementary Table 1. The HWE (Hardy-Weinberg equilibrium) tests showed the p value below 0.05 at HLD56 (Table 1). Therefore we used the validation analysis by program HWE (version 1.10) [10] observed three *p* values below 0.05 at HLD56, HLD81, and HLD118 loci, respectively. And these two independent analysts with high efficiency analyzed the HWE tests of 30 InDels in Bai ethnic group. After the Bonferroni correction (significance level, 0.0017), no deviations from HWE tests were observed at the 30 Indel loci of the Bai group. The observed heterozygosity (Ho) values ranged from 0.1520 at HLD118 locus to 0.5680 at HLD131 locus, while the expected heterozygosity (He) ranged from 0.1927 at HLD118 locus to 0.4997 at HLD136 locus, and the polymorphic information content (PIC) ranged from 0.1741 at HLD118 locus to 0.3749 at HLD136 locus. We found the highest power of exclusion (PE) (0.2542) at HLD131 locus and the lowest PE (0.0181) at HLD118 locus. The DP values of selected loci were in the range from 0.3100 (HLD118) to 0.6533 (HLD56). The highest value of typical paternity index (TPI) was 1.1574 (HLD131) and the lowest was 0.5896 (HLD118).

**Table 1:** Allele frequency distribution and forensic statistical parameters of the 30 Indel loci in Chinese Bai ethnic group (n=125)

HLD	rs#	DIP-	DIP+	Ho	He	*p*	TPI	PIC	PE	DP
6	1610905	0.5200	0.4800	0.4640	0.4992	0.4054	0.9328	0.3746	0.1579	0.6403
39	17878444	0.8320	0.1680	0.3040	0.2796	0.5617	0.7184	0.2405	0.0652	0.4449
40	2307956	0.3840	0.6160	0.4000	0.4731	0.0932	0.8333	0.3612	0.1139	0.6331
45	2307959	0.3920	0.6080	0.4640	0.4767	0.7441	0.9328	0.3631	0.1579	0.6177
48	28369942	0.6360	0.3640	0.4560	0.4630	0.8424	0.9191	0.3558	0.1518	0.6071
56	2308292	0.4360	0.5640	0.3920	0.4918	0.0228	0.8224	0.3709	0.1091	0.6533
58	1610937	0.6160	0.3840	0.4800	0.4731	0.9107	0.9615	0.3612	0.1706	0.6075
64	1610935	0.1320	0.8680	0.2160	0.2292	0.7085	0.6378	0.2029	0.0343	0.3752
67	1305056	0.3280	0.6720	0.4320	0.4408	0.8114	0.8803	0.3437	0.1346	0.5929
70	2307652	0.3560	0.6440	0.4560	0.4585	0.9219	0.9191	0.3534	0.1518	0.6026
77	1611048	0.5600	0.4400	0.5280	0.4928	0.4576	1.0593	0.3714	0.2132	0.6026
81	17879936	0.1400	0.8600	0.1840	0.2408	0.1314	0.6127	0.2118	0.0256	0.3740
83	2308072	0.6200	0.3800	0.5520	0.4712	0.0772	1.1161	0.3602	0.2372	0.5661
84	3081400	0.3000	0.7000	0.3760	0.4200	0.3010	0.8013	0.3318	0.1000	0.5839
88	8190570	0.4720	0.5280	0.5120	0.4984	0.7959	1.0246	0.3742	0.1982	0.6172
92	17174476	0.5360	0.4640	0.4480	0.4974	0.2504	0.9058	0.3737	0.1459	0.6444
93	2307570	0.4600	0.5400	0.4240	0.4968	0.0944	0.8681	0.3734	0.1292	0.6511
97	17238892	0.6840	0.3160	0.3920	0.4323	0.3431	0.8224	0.3389	0.1091	0.5938
99	2308163	0.2000	0.8000	0.3040	0.3200	0.6790	0.7184	0.2688	0.0652	0.4854
101	2307433	0.5520	0.4480	0.4480	0.4946	0.2774	0.9058	0.3723	0.1459	0.6415
111	1305047	0.8880	0.1120	0.1920	0.1989	0.8293	0.6188	0.1791	0.0276	0.3356
114	2307581	0.8160	0.1840	0.3200	0.3003	0.6521	0.7353	0.2552	0.0721	0.4667
118	16438	0.1080	0.8920	0.1520	0.1927	0.2408	0.5896	0.1741	0.0181	0.3100
122	8178524	0.7600	0.2400	0.3680	0.3648	0.9679	0.7911	0.2983	0.0956	0.5297
124	6481	0.4640	0.5360	0.4800	0.4974	0.6643	0.9615	0.3737	0.1706	0.6318
125	16388	0.5480	0.4520	0.5360	0.4954	0.3878	1.0776	0.3727	0.2210	0.6004
128	2307924	0.7480	0.2520	0.3600	0.3770	0.6697	0.7813	0.3059	0.0914	0.5426
131	1611001	0.5720	0.4280	0.5680	0.4896	0.0875	1.1574	0.3698	0.2542	0.5737
133	2067235	0.6120	0.3880	0.5040	0.4749	0.5429	1.0081	0.3621	0.1910	0.5979
136	16363	0.4880	0.5120	0.4480	0.4997	0.2297	0.9058	0.3749	0.1459	0.6467

HLD, human locus deletion/insertion polymorphism; DIP-, frequency of short allele; DIP+, frequency of long allele; Ho, observed heterozygosity; He, expected heterozygosity; *p*, *p*-value for Hardy-Weinberg equilibrium; TPI, typical paternity index; PIC, polymorphic information content; PE, power of exclusion; DP, discrimination power.

### Population structure

**Figure 2:**
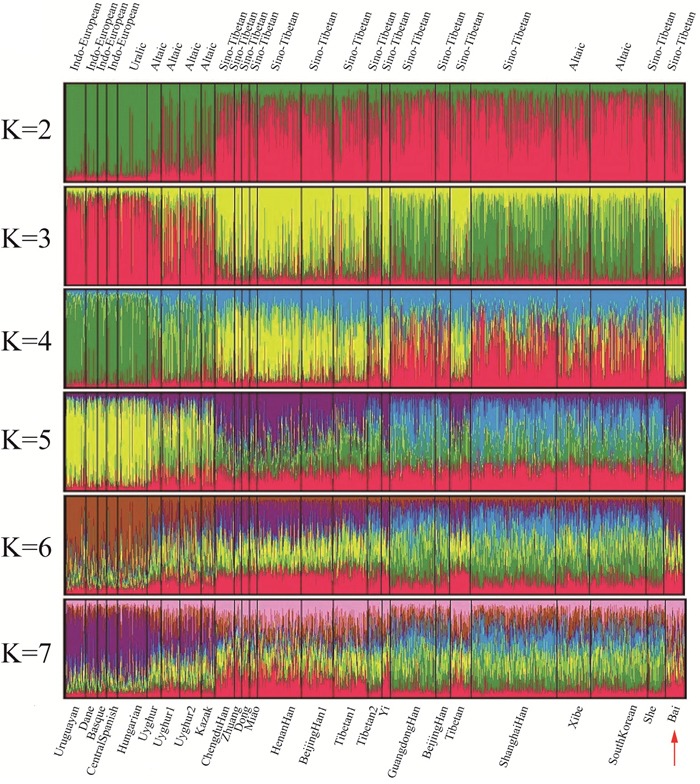
Clustering structure for the full-loci dataset assuming *K*= 2-7 of the Bai group with other groups Population names were labeled beneath while language names on top.

Together with 25 referenced populations i.e. Guangdong Han [11], Shanghai Han [12], Yi [13], Xibe [14], South Korean [15], She [16], Beijing Han, Tibetan, Kazak, Uyghur [17], Dane [18], Hungarian [19], Basque, Central Spanish [20], Uruguayan [21], Uyghur1, Zhuang, Dong, Miao, Chengdu Han, Tibetan1 [22], Henan Han, Beijing Han1, Uyghur2, Tibetan2 [23], the population structure of Bai ethnic group was performed by the structure program. In the determination of *K* value (Supplementary Figure 1), the results of L(*K*) showed that *K*=3, 4 depicted a plateau obviously. As the maximum value of Delta *K* was observed at *K*=3, it demonstrated that *K*=3 met the requirements as mentioned by Porras [24]. At *K*=3, the 26 populations were clarified into 3 geographic patterns clearly as shown in Figure 2. The 4 Eurasian groups (Uyghur, Uyghur1, Uyghur2, and Kazak), Uruguayan and 4 European populations were composed of nearly entire red component, by contrast, the 17 East Asian populations were composed of yellow, green, and red components by different proportions.

### Principal component analysis

**Figure 3:**
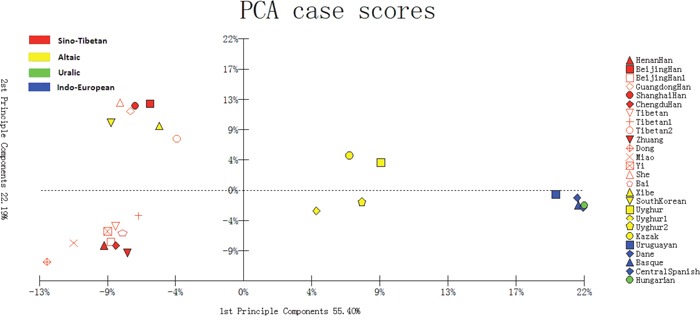
PCA analysis based on 30 Indel loci of the studied Bai group and referenced 25 groups

In Figure 3, the allele frequencies of 30 Indel loci of the Bai ethnic group in the study along with referenced 25 populations were utilized to perform a PCA plot. As shown in Figure 3, the first component accounted for 55.40% and the second 22.19%. After aggregation, the two principle components occupied the 77.59% of total variance.

### Population differentiations

We employed the Analysis of molecular variance method to compare the studied Bai group with 25 reported populations mentioned above. The *p* values of *Fst* distances were shown in Table 2. The minimum and maximum numbers of significant difference observed at 30 loci were 2 (Bai and Yi, Bai and Tibetan1), and 22 (Bai and Hungarian), respectively. In Supplementary Table 2, we calculated the *D_A_* values among 26 populations, ranging from 0.0006 (Guangdong Han and Shanghai Han, Henan Han and Beijing Han1) to 0.0358 (Dong and Hungarian). Among Bai and other 25 reported populations, the minimum was 0.0012 (Bai and Tibetan1) whereas the maximum was 0.0238 (Bai and Basque).

**Table 2:** The *p* values of pairwise InDel loci between Chinese Bai group and referenced populations at 30 Indel loci

Loci	Guangdong Han	Shanghai Han	Beijing Han	Beijing Han1	Henan Han	Chengdu Han	Yi	Xibe	South Korean	She	Tibetan	Tibetan1	Tibetan2	Zhuang	Dong	Miao	Uyghur	Uyghur1	Uyghur2	Kazak	Dane	Basque	Central Spanish	Hungarian	Uruguayan
HLD6	0.6481	1.0000	0.7693	1.0000	1.0000	1.0000	0.8221	0.2043	0.2532	0.0919	1.0000	1.0000	1.0000	1.0000	0.4897	**0.0420**	0.1984	0.1222	0.1075	0.0645	0.5367	0.4614	1.0000	0.6100	**0.0235**
HLD39	**0.0000**	**0.0000**	**0.0000**	0.2024	0.1036	0.4995	0.3138	**0.0000**	**0.0000**	**0.0000**	**0.0000**	0.6432	0.0645	0.2532	**0.0215**	0.2297	**0.0000**	**0.0010**	**0.0000**	**0.0000**	**0.0000**	**0.0000**	**0.0000**	**0.0000**	**0.0000**
HLD40	**0.0000**	**0.0000**	**0.0000**	0.0938	0.1916	0.3500	0.4946	**0.0000**	**0.0000**	**0.0000**	**0.0000**	1.0000	0.2199	1.0000	**0.0469**	1.0000	**0.0000**	**0.0000**	**0.0078**	**0.0000**	**0.0029**	0.0782	**0.0000**	**0.0020**	**0.0000**
HLD45	1.0000	1.0000	0.4614	0.8749	1.0000	0.7155	0.8221	1.0000	1.0000	0.3275	0.1437	0.2688	0.1603	1.0000	0.6413	**0.0323**	0.3314	1.0000	1.0000	**0.0156**	**0.0499**	0.0860	0.2209	**0.0039**	0.0968
HLD48	1.0000	1.0000	0.5396	0.4878	0.3480	1.0000	1.0000	0.3627	1.0000	1.0000	0.0987	0.7107	**0.0459**	0.1720	0.3509	1.0000	0.6393	**0.0440**	**0.0010**	0.1066	**0.0000**	**0.0078**	**0.0000**	**0.0068**	**0.0000**
HLD56	0.0948	0.2639	0.6012	0.5748	0.4409	0.4379	0.4937	0.6432	0.5640	1.0000	0.3011	1.0000	0.2131	1.0000	0.1466	0.5914	0.8231	1.0000	0.4145	1.0000	0.1525	0.6061	**0.0010**	**0.0029**	1.0000
HLD58	**0.0303**	1.0000	0.7107	0.3206	1.0000	1.0000	0.0665	**0.0284**	0.2180	1.0000	0.6833	0.1378	**0.0098**	0.4145	**0.0196**	0.1779	0.5455	0.1300	1.0000	1.0000	**0.0000**	**0.0000**	**0.0039**	**0.0010**	0.0987
HLD64	0.4487	0.2845	1.0000	**0.0352**	0.1701	0.1007	1.0000	**0.0020**	1.0000	1.0000	0.5689	0.9130	1.0000	0.8299	0.3910	0.7107	**0.0000**	**0.0000**	**0.0000**	**0.0000**	**0.0000**	**0.0000**	**0.0000**	**0.0000**	**0.0000**
HLD67	0.1965	1.0000	1.0000	1.0000	0.2981	0.3275	0.4761	0.3548	1.0000	**0.0098**	0.0909	0.1378	0.0811	0.2659	**0.0059**	**0.0108**	0.7146	0.1300	**0.0000**	**0.0401**	0.1476	**0.0000**	0.5924	**0.0352**	**0.0332**
HLD70	**0.0489**	**0.0313**	**0.0039**	0.1945	0.7077	1.0000	1.0000	1.0000	1.0000	**0.0020**	**0.0049**	**0.0098**	0.1672	0.4467	0.3216	0.0743	0.4976	0.5513	1.0000	0.3842	**0.0108**	1.0000	1.0000	**0.0127**	1.0000
HLD77	1.0000	0.3011	0.0528	0.2239	0.4311	1.0000	0.1799	0.0655	1.0000	0.5044	1.0000	1.0000	0.2845	0.1222	0.1789	0.1241	1.0000	1.0000	1.0000	1.0000	**0.0020**	0.0909	0.8250	1.0000	0.3128
HLD81	**0.0430**	0.2239	1.0000	0.8065	1.0000	0.1720	1.0000	1.0000	1.0000	0.0714	1.0000	1.0000	1.0000	**0.0029**	0.3509	0.2688	**0.0010**	**0.0000**	**0.0000**	**0.0000**	**0.0000**	**0.0000**	**0.0000**	**0.0000**	**0.0000**
HLD83	1.0000	1.0000	**0.0039**	1.0000	1.0000	1.0000	1.0000	1.0000	1.0000	**0.0244**	0.4653	1.0000	**0.0156**	1.0000	0.1290	1.0000	1.0000	0.3431	0.1857	1.0000	**0.0000**	**0.0000**	**0.0049**	0.0528	0.1281
HLD84	**0.0020**	0.2102	1.0000	1.0000	0.3988	0.0587	1.0000	**0.0059**	0.1114	**0.0068**	1.0000	1.0000	1.0000	**0.0166**	**0.0020**	**0.0499**	**0.0469**	1.0000	0.5200	1.0000	**0.0000**	**0.0293**	0.0841	**0.0010**	**0.0000**
HLD88	1.0000	1.0000	0.0772	1.0000	0.6080	**0.0293**	0.6403	0.2835	1.0000	0.5191	0.0508	**0.0186**	**0.0088**	1.0000	0.1417	0.3519	0.4555	1.0000	1.0000	0.3109	0.3460	**0.0264**	0.0557	0.3695	0.1437
HLD92	0.5415	1.0000	1.0000	1.0000	0.2825	0.0645	0.6540	1.0000	0.2952	1.0000	0.7537	1.0000	0.4311	0.5738	1.0000	0.1642	0.3460	1.0000	1.0000	0.0850	1.0000	0.6618	1.0000	0.3079	0.3998
HLD93	1.0000	1.0000	1.0000	0.1916	1.0000	0.2678	0.4526	**0.0401**	0.0821	**0.0264**	0.0948	1.0000	0.3079	0.2698	0.2600	1.0000	0.3607	1.0000	1.0000	0.4330	0.3226	0.5367	1.0000	1.0000	0.6198
HLD97	0.2991	1.0000	0.2385	1.0000	0.7009	0.4663	**0.0342**	0.2278	1.0000	0.3959	1.0000	0.1085	0.0860	0.6413	0.1554	0.7077	0.1163	0.3324	0.4907	0.4751	**0.0000**	**0.0000**	**0.0000**	**0.0000**	**0.0000**
HLD99	**0.0029**	**0.0039**	0.8954	**0.0137**	**0.0049**	**0.0205**	1.0000	0.2219	**0.0000**	**0.0117**	0.2815	0.7664	1.0000	0.0958	1.0000	1.0000	**0.0000**	0.1398	**0.0068**	**0.0000**	**0.0000**	**0.0010**	**0.0000**	**0.0000**	**0.0000**
HLD101	1.0000	1.0000	0.6373	0.3118	0.5777	1.0000	1.0000	0.4526	1.0000	0.2551	0.8612	0.5572	0.4409	1.0000	0.2199	0.2248	**0.0020**	0.7214	0.4213	0.0763	0.1584	1.0000	0.3480	0.6852	0.1926
HLD111	1.0000	0.2121	0.6237	0.6833	1.0000	1.0000	1.0000	1.0000	0.0518	1.0000	1.0000	0.5083	0.1251	0.3441	0.5220	1.0000	**0.0000**	**0.0010**	**0.0000**	**0.0000**	**0.0000**	**0.0000**	**0.0000**	**0.0000**	**0.0000**
HLD114	0.0763	**0.0020**	**0.0078**	**0.0010**	**0.0156**	**0.0049**	**0.0059**	**0.0000**	**0.0020**	0.0723	**0.0186**	0.3539	**0.0010**	0.3001	1.0000	1.0000	**0.0000**	**0.0000**	**0.0000**	**0.0000**	**0.0000**	**0.0049**	**0.0000**	**0.0000**	**0.0000**
HLD118	0.2727	**0.0166**	0.1965	0.6256	0.1085	0.1212	0.0733	0.5005	**0.0010**	0.4829	0.0792	1.0000	0.6022	1.0000	**0.0108**	1.0000	**0.0000**	**0.0000**	**0.0000**	**0.0000**	**0.0000**	**0.0000**	**0.0000**	**0.0000**	**0.0000**
HLD122	0.2307	1.0000	1.0000	0.5240	1.0000	1.0000	0.1535	0.5269	0.7058	**0.0137**	**0.0029**	1.0000	**0.0068**	**0.0293**	1.0000	0.0606	**0.0020**	**0.0000**	**0.0010**	**0.0108**	**0.0039**	0.0753	**0.0000**	**0.0000**	**0.0000**
HLD124	1.0000	1.0000	1.0000	0.3500	0.1515	1.0000	1.0000	0.2483	1.0000	1.0000	1.0000	1.0000	1.0000	1.0000	1.0000	0.1838	1.0000	0.1271	0.6413	0.4858	1.0000	**0.0098**	**0.0459**	**0.0137**	**0.0156**
HLD125	0.0841	**0.0020**	1.0000	0.0987	**0.0010**	0.5024	0.0665	0.3646	**0.0059**	0.3226	1.0000	0.5034	1.0000	1.0000	0.5347	1.0000	0.2385	1.0000	0.0811	1.0000	0.0655	1.0000	1.0000	**0.0332**	1.0000
HLD128	**0.0000**	**0.0000**	**0.0049**	0.4399	**0.0284**	0.1026	1.0000	**0.0020**	**0.0000**	**0.0000**	1.0000	0.2102	**0.0059**	**0.0381**	1.0000	1.0000	0.3118	**0.0000**	**0.0000**	1.0000	0.4477	1.0000	**0.0068**	**0.0000**	**0.0010**
HLD131	**0.0147**	**0.0186**	0.3285	**0.0244**	0.4633	0.0655	1.0000	**0.0010**	0.0811	0.0606	0.1554	0.1828	1.0000	**0.0039**	**0.0020**	0.4643	0.1711	0.2610	0.3822	1.0000	0.0518	0.6725	0.1975	**0.0000**	**0.0293**
HLD133	0.4233	0.6706	1.0000	1.0000	0.5308	1.0000	1.0000	1.0000	1.0000	0.0684	0.2806	0.2043	0.5103	0.1574	0.3128	0.0909	1.0000	1.0000	1.0000	0.2806	**0.0000**	**0.0010**	**0.0000**	**0.0000**	**0.0020**
HLD136	0.4067	1.0000	0.3910	0.1916	0.7371	0.4516	1.0000	0.7644	0.0665	0.1349	0.7058	1.0000	0.0841	0.5073	1.0000	**0.0020**	1.0000	0.4927	1.0000	1.0000	1.0000	**0.0010**	0.6129	0.8592	0.3910

**Significant differences (*p* < 0.05) were in bold**

### Phylogenetic reconstruction

**Figure 4:**
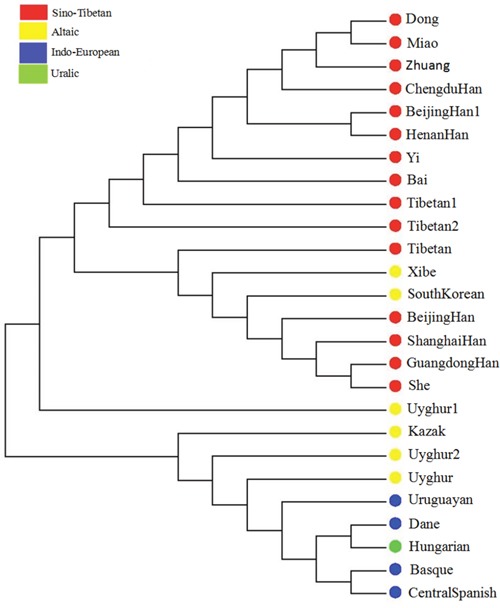
Phylogenetic tree constructed by the agglomerative clustering (Neighbor Joining) method based on the *D_A_* distances between the 26 groups

To analyze the genetic background of Bai group, we drew a Neighbor Joining (NJ) Tree [25] based on the *D_A_* genetic distances (Supplementary Table 2). The NJ tree showed two main clusters (Figure 4). One comprised the Uyghur1 and 17 East Asian populations (She, Guangdong Han, Shanghai Han, Beijing Han, South Korean, Xibe, Tibetan, Tibetan2, Tibetan1, Bai, Yi, Henan Han, Beijing Han1, Chengdu Han, Zhuang, Miao and Dong populations). And the other was composed of the Kazak, Uyghur2, Uyghur, Uruguayan and other European groups (Dane, Hungarian, Basque, and Central Spanish).

## DISCUSSION

### Linkage disequilibrium analysis

If no relevance exists between two loci from the same chromosome or two random chromosomes, in other words, they are suitable for forensic applications as independent loci [26]. The LD analysis indicated no significant LD (*r^2^*< 0.8) among the 30 loci. We observed the LD analysis of studied Bai group and Chinese Yi group previously reported [27], those two studies obviously verified no linkage among 30 Indel loci, therefore, those loci could be used as independent markers for forensic and population genetic analysis.

### Forensic parameters

In this research, the cumulative probability of exclusion (CPE) and combined discrimination power (CDP) were 0.9859 and 0.9999999999887, respectively. The CPE values were 0.9861, 0.9968, 0.9957, 0.9975, 0.9884, 0.9900 in the previously published Dong, Uyghur1 [22], Tibetan, Uyghur [17], Taiwanese, Poles [28], respectively. And we could observe that the CPE of 30 Indel loci in various populations changed within a narrow scope. Collectively, the CPE values using the kit were relatively lower and could not reach a high level of exclusion in forensic paternity cases compared to STR genetic markers [29, 30]. Nonetheless, the CDP values had the ability to provide considerable level of discrimination when the kit came to forensic identification cases. According to the formula calculating PIC [31], we established the PIC calculation model of diallelic markers: PIC=2*p*-4*p*^2^+4*p*^3^-2*p*^4^, *p* represented the frequency of random InDel allele (+ or -). PIC value was expected to range from 0 to 0.5. Accordingly, it indicated that the forensic parameter PIC would be less than 0.5 for diallelic genetic markers, for example InDels and SNPs [32], much lower than STRs [33]. To be concluded, we could utilize the kit as supplementary mean for forensic cases tested by autosomal STRs.

### Population structure

Individuals of different populations far from each other (for example, Asian and European) always had diverse membership coefficient in deductive clusters. In the present study, at *K*=3, the 10 East Asian groups (Chengdu Han, Zhuang, Dong, Miao, Henan Han, Beijing Han1, Tibetan1, Yi, Tibetan, and Bai) were all constituted of different components by an uniform proportion, which indicated the Bai group's similar membership to East Asian populations. Compared to the 17 East Asian groups, constructions of the 4 Eurasian groups (Uyghur, Uyghur1, Uyghur2, and Kazak) were more semblable with the Uruguayan and European groups [34]. Based on the instruction of structure program [35], the populations sharing similar structure were close in memberships. Therefore, through structure analysis on raw data of 30 Indel loci, the Bai ethnic group was homogeneous with other Asian groups.

### Principal component analysis

As shown in Figure 3, the European groups and Uruguayan distributed in the right; Chinese 16 populations and South Korean in the left; Uyghur1, Kazak, Uyghur2, and Uyghur groups from China in the middle of them. The result of PCA corresponded with structure analysis and geographic location. The studied Bai group, together with Henan Han, Beijing Han1, Chengdu Han, Dong, Miao, Zhuang, Yi, Tibetan1 and Tibetan groups clustered in the lower left quadrant, which indicated their close genetic relationships.

### Population differentiations

The values of *D_A_* genetic distance were consistent with the geographic locations of these populations. Supposing threshold value was 0.05, we could observe the significant differences between the Bai group and 25 referenced groups. Compared with East Asian groups and Eurasian groups, Central Spanish, Uruguayan, Dane, Basque and Hungarian groups were found had more differences with Bai group. Significant differences between the Bai group and all East Asian and Eurasian groups were found at less than 12 loci, whereas the Bai group and Uruguayan as well as European groups were more than 17 loci. For geographical concerns, the results corresponded with the geographic distribution of the 26 groups.

In the aspects of 30 loci, the highest ethnic diversity was found at HLD114 locus which reflected significant differences between the studied Bai group and other 19 groups. By contrast, no significant differences were observed at HLD92 locus between the Bai group and other 25 groups. Similar to STRs [36, 37], the ability of Indels to distinguish ethnic groups was at various levels. Along with more typing data of different populations at novel Indel loci, evolution in human history would be investigated comprehensively and detailedly.

### Phylogenetic reconstruction

The results of the phylogenetic reconstruction were roughly in line with population differentiation. Previously, we had reported that Bai ethnic group was close to Southern Chinese Han, Changsha Han, and Guangdong Han populations within the scope of mitochondrial DNA [38]. It demonstrated that Bai ethnic group in Chinese Yunnan Province had multi origins, for example, Chinese Han groups, Yi group and Tibetan group, etc. In history, descents of different ethnic groups integrated into Yunnan Bai group including Diqiang system from Northern China, Pu system from Southern China and Chinese Han [39] (http://www.china.org.cn/e-groups/shaoshu/shao-2-bai.htm). Diqiang and Pu systems were both ancient nations in China dating back to 1000 BC. Ancient Diqiang was the origin of Tibetan, and ancient Pu was the origin of Dai, Blang, De'ang and Va ethnic minorities [40]. Hence, our study illustrated Bai ethnic group in Yunnan province was not only close to Chinese Han in relationship, but also integrated into the Tibetan and other ethnic groups. In summary, the inference focusing on the association between studied Bai group and Chinese Han population nationwide, Yi, Tibetan group and other ethnic groups was supported by the studies of population structure, principal component analysis, population differentiations, and phylogenetic reconstruction, which was consistent with population migration and cultural exchange in history. Studies on Bai ethnic group utilizing STR as genetic markers [41, 42] also indicated a close relationship between Bai group and Han populations nationwide.

## MATERIALS AND METHODS

### Samples and DNA extraction

We collected bloodstain samples from 125 unrelated healthy Bai individuals living in Dali Bai Autonomous Prefecture in Yunnan province with informed consent. In this study, donors should have ancestors living in Dali for over three generations and have no significant migration in their family history. The collection process follows the human and ethical research principles of Xi'an Jiaotong University Health Science Center, China. The Chelex-100 method was used to extract genomic DNA from bloodstain samples as described by Walsh [43].

### PCR amplification and genotyping for InDels

In this study, we employed Investigator DIPplex kit for InDels genotyping including 30 InDel loci plus a sex-determining locus Amelogenin, which has been validated before [8, 44]. The PCR amplification conducted in a GeneAmp PCR System 9700 Thermal Cycler (Applied Biosystems, Foster City, CA, USA) in accordance with the manufacturer's instruction. After that, the ABI 3130 Genetic Analyzer (Applied Biosystems, Foster City, CA, USA) was utilized to perform electrophoresis in the conditions described in the manufacturer's recommendations. With the BTO 550 (Qiagen, Hilden, Germany) as internal lane standard, we could determine the fragment sizing. We used the GeneMapperID software v3.2 (Applied Biosystems, Foster City, CA, USA) to identify the alleles.

### Quality control

The study was conducted following ISFG recommendations as Schneider described in the aspects of DNA polymorphisms [45].

### Statistical analyses

The modified PowerStates (version 1.2) spreadsheet (Promega, Madison, WI, USA) was used to calculate the allele frequencies, forensic parameters and the exact chi-square test for the HWE of 30 InDels. The forensic parameters included Ho, PE, PIC, DP, and TPI. He values were calculated by the formula, $\text{He}=\frac{\text{n}}{\text{n}-1}\left(1-\sum_{\text{i}=1}^{\text{n}}{\text{P}}_{\text{i}}^{2}\right)$ [46]. We utilized the SNP Analyzer version 2.0 (Istech, South Korea) [47] to perform LD analysis. We estimated *Fst* and *p* values between pairwise populations by Arlequin (version 3.0) [48] to embody the variances in allele frequencies of different populations. To analyze the population structure, we used the Structure program (version 2.2) [49] to estimate the membership coefficients. The Ln Pr(X|*K*) [50] and Delta *K* [51] were calculated to determine the most appropriate *K* value. On the basis of allele frequencies, principal component analysis (PCA) was carried out in MATLAB 2007a (MathWorks Inc., USA). For phylogenetic reconstruction, we utilized the *D_A_* distance and employed phylogenetic analysis (DISPAN) program [52].

## CONCLUSION

In conclusion, the combination of 30 Indel loci provided a relatively low level CPE (0.9859) and a high level of CDP (0.9999999999887). Research of population genetics on population structure, principal component analysis, population differentiations, and phylogenetic reconstruction of Bai group based on 30 Indel loci supplementally supported that the Bai group had close relationships with certain ethnic groups from China, for example Tibetan and Yi group, consistent with the analysis by STR markers and history migration. With more genotyping profiles of various ethnic groups, we could have a comprehensive understanding of population migration and ancestry origin in China.

## SUPPLEMENTARY FIGURE AND TABLES






