# Supplementary material for: Genetic variation and forensic efficiency of autosomal insertion/deletion polymorphisms in Chinese Bai ethnic group: phylogenetic analysis to other populations
Source: Oncotarget. 2017 Apr 17;8(24):39582–91. doi: 10.18632/oncotarget.17137 (PMC5503634; doi:10.18632/oncotarget.17137)
Supplement: Supplementary file 1 [file oncotarget-08-39582-s001.pdf]

Genetic variation and forensic efficiency of autosomal insertion/deletion polymorphisms in Chinese Bai ethnic group: phylogenetic analysis to other populations

SUPPLEMENTARY FIGURE AND TABLES

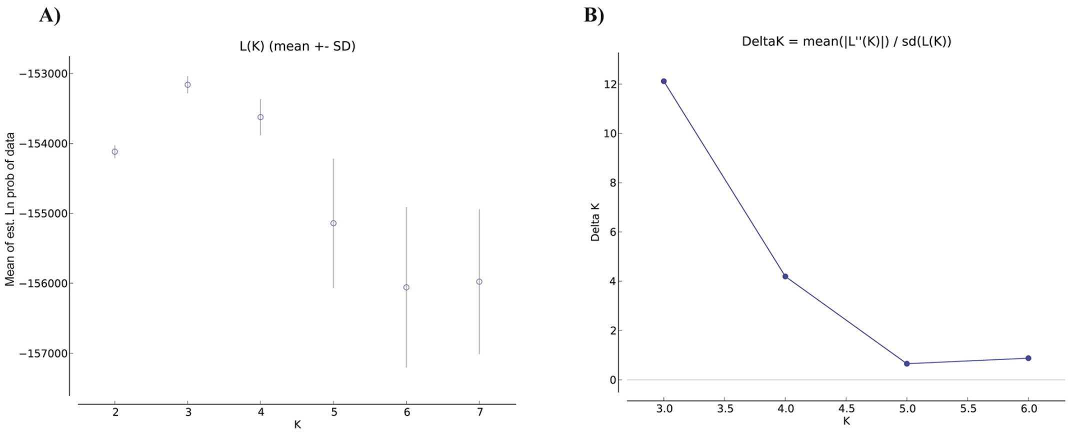

Supplementary Figure 1: Diagram form of the estimated probability of data at *K*=2-7. (A) The variances of the estimated probability value at *K*=2-7. We could observed a plateau at *K*=3, 4. **(B)** The maximum of delta *K* value calculated was found at *K*=3.

Supplementary Table 1: The raw data of 30 Indels from 125 unrelated healthy Bai individuals

See Supplementary File 1

Supplementary Table 2: The values of DA of pairwise populations among Chinese Bai group and referenced populations

See Supplementary File 2
