# Supplementary material for: Genetic variation and forensic efficiency of autosomal insertion/deletion polymorphisms in Chinese Bai ethnic group: phylogenetic analysis to other populations
Source: Oncotarget. 2017 Apr 17;8(24):39582–91. doi: 10.18632/oncotarget.17137 (PMC5503634; doi:10.18632/oncotarget.17137)
Supplement: Supplementary file 2 [file oncotarget-08-39582-s002.doc]

**Supplementary Table 1. The raw data of 30 Indels** from 125 unrelated healthy Bai individuals.

| Sample ID | HLD  6 | | | | | | | | | HLD  39 | | | | | | | | | | | HLD  40 | | | | | | | | | | HLD  45 | | | | | | | | | | HLD  48 | | | | | | | | HLD  56 | | | | | | | | HLD  58 | | | | | | | | HLD  64 | | | | | | | | | HLD  67 | | | | | | | | | HLD  70 | | | | | | | | | HLD  77 | | | | | | | | | HLD  81 | | | | | | | | | HLD  83 | | | | | | | | | HLD  84 | | | | | | | | | HLD  88 | | | | | | | | | HLD  92 | | | | | | | | | HLD  93 | | | | | | | | | HLD  97 | | | | | | | | | HLD  99 | | | | | | | | | HLD  101 | | | | | | | | HLD  111 | | | | | | | | HLD  114 | | | | | | | | HLD  118 | | | | | | | | HLD  122 | | | | | | | | HLD  124 | | | | | | | | HLD  125 | | | | | | | | HLD  128 | | | | | | | | HLD  131 | | | | | | | | HLD  133 | | | | | | | | HLD  136 | | | | |
| --- | --- | --- | --- | --- | --- | --- | --- | --- | --- | --- | --- | --- | --- | --- | --- | --- | --- | --- | --- | --- | --- | --- | --- | --- | --- | --- | --- | --- | --- | --- | --- | --- | --- | --- | --- | --- | --- | --- | --- | --- | --- | --- | --- | --- | --- | --- | --- | --- | --- | --- | --- | --- | --- | --- | --- | --- | --- | --- | --- | --- | --- | --- | --- | --- | --- | --- | --- | --- | --- | --- | --- | --- | --- | --- | --- | --- | --- | --- | --- | --- | --- | --- | --- | --- | --- | --- | --- | --- | --- | --- | --- | --- | --- | --- | --- | --- | --- | --- | --- | --- | --- | --- | --- | --- | --- | --- | --- | --- | --- | --- | --- | --- | --- | --- | --- | --- | --- | --- | --- | --- | --- | --- | --- | --- | --- | --- | --- | --- | --- | --- | --- | --- | --- | --- | --- | --- | --- | --- | --- | --- | --- | --- | --- | --- | --- | --- | --- | --- | --- | --- | --- | --- | --- | --- | --- | --- | --- | --- | --- | --- | --- | --- | --- | --- | --- | --- | --- | --- | --- | --- | --- | --- | --- | --- | --- | --- | --- | --- | --- | --- | --- | --- | --- | --- | --- | --- | --- | --- | --- | --- | --- | --- | --- | --- | --- | --- | --- | --- | --- | --- | --- | --- | --- | --- | --- | --- | --- | --- | --- | --- | --- | --- | --- | --- | --- | --- | --- | --- | --- | --- | --- | --- | --- | --- | --- | --- | --- | --- | --- | --- | --- | --- | --- | --- | --- | --- | --- | --- | --- | --- | --- | --- | --- | --- | --- | --- | --- | --- | --- | --- | --- | --- | --- | --- | --- | --- | --- |
| 121 | | | 1 | | 1 | | | | | 2 | | | | | | 2 | | | | 2 | | | | | | 1 | | | | 1 | | | | | | 1 | | | | 2 | | | | | 2 | | | 2 | | | | | 1 | | | 2 | | | | | 1 | | | 2 | | | | | 1 | | | 2 | | | | | | 1 | | | 2 | | | | | | 1 | | | 2 | | | | | | 1 | | | 1 | | | | | | 1 | | | 2 | | | | | | 1 | | | 1 | | | | | | 1 | | | 2 | | | | | | 1 | | | 1 | | | | | | 1 | | | 2 | | | | | | 1 | | | 2 | | | | | | 2 | | | 2 | | | | | | 1 | | | 1 | | | | | 1 | | | 2 | | | | | 2 | | | 1 | | | | | 1 | | | 1 | | | | | 1 | | | | 2 | | | | 2 | | | | 1 | | | | 1 | | | | 1 | | | | 1 | | | | 2 | | | | 2 | | | | 2 | | | | 1 | | | | 2 | | | | 2 | | | | 2 | | | | 2 | |
| 122 | | | 2 | | 1 | | | | | 2 | | | | | | 1 | | | | 2 | | | | | | 1 | | | | 2 | | | | | | 1 | | | | 2 | | | | | 1 | | | 1 | | | | | 1 | | | 2 | | | | | 2 | | | 2 | | | | | 2 | | | 1 | | | | | | 1 | | | 2 | | | | | | 2 | | | 2 | | | | | | 1 | | | 1 | | | | | | 1 | | | 2 | | | | | | 1 | | | 1 | | | | | | 1 | | | 1 | | | | | | 1 | | | 2 | | | | | | 2 | | | 2 | | | | | | 1 | | | 2 | | | | | | 2 | | | 1 | | | | | | 1 | | | 1 | | | | | 1 | | | 2 | | | | | 2 | | | 2 | | | | | 1 | | | 2 | | | | | 1 | | | | 2 | | | | 1 | | | | 2 | | | | 1 | | | | 2 | | | | 1 | | | | 2 | | | | 2 | | | | 2 | | | | 2 | | | | 2 | | | | 2 | | | | 1 | | | | 1 | |
| 123 | | | 1 | | 1 | | | | | 2 | | | | | | 2 | | | | 2 | | | | | | 2 | | | | 1 | | | | | | 1 | | | | 2 | | | | | 2 | | | 1 | | | | | 1 | | | 2 | | | | | 2 | | | 2 | | | | | 1 | | | 2 | | | | | | 1 | | | 1 | | | | | | 1 | | | 2 | | | | | | 2 | | | 2 | | | | | | 2 | | | 2 | | | | | | 1 | | | 2 | | | | | | 1 | | | 1 | | | | | | 1 | | | 2 | | | | | | 1 | | | 2 | | | | | | 1 | | | 2 | | | | | | 1 | | | 1 | | | | | | 1 | | | 2 | | | | | 1 | | | 2 | | | | | 2 | | | 2 | | | | | 2 | | | 2 | | | | | 2 | | | | 2 | | | | 1 | | | | 2 | | | | 1 | | | | 2 | | | | 1 | | | | 2 | | | | 1 | | | | 2 | | | | 1 | | | | 2 | | | | 1 | | | | 2 | | | | 2 | |
| 124 | | | 2 | | 1 | | | | | 2 | | | | | | 2 | | | | 2 | | | | | | 1 | | | | 2 | | | | | | 2 | | | | 2 | | | | | 2 | | | 2 | | | | | 2 | | | 2 | | | | | 2 | | | 1 | | | | | 1 | | | 1 | | | | | | 1 | | | 2 | | | | | | 1 | | | 2 | | | | | | 2 | | | 2 | | | | | | 1 | | | 1 | | | | | | 2 | | | 1 | | | | | | 1 | | | 1 | | | | | | 1 | | | 1 | | | | | | 1 | | | 2 | | | | | | 1 | | | 2 | | | | | | 2 | | | 1 | | | | | | 1 | | | 2 | | | | | 1 | | | 2 | | | | | 1 | | | 2 | | | | | 2 | | | 2 | | | | | 1 | | | | 2 | | | | 2 | | | | 2 | | | | 1 | | | | 2 | | | | 1 | | | | 2 | | | | 2 | | | | 2 | | | | 2 | | | | 2 | | | | 1 | | | | 2 | | | | 1 | |
| 125 | | | 2 | | 2 | | | | | 2 | | | | | | 2 | | | | 1 | | | | | | 1 | | | | 2 | | | | | | 1 | | | | 2 | | | | | 2 | | | 2 | | | | | 1 | | | 2 | | | | | 2 | | | 1 | | | | | 1 | | | 1 | | | | | | 1 | | | 2 | | | | | | 1 | | | 2 | | | | | | 2 | | | 1 | | | | | | 1 | | | 2 | | | | | | 1 | | | 2 | | | | | | 2 | | | 2 | | | | | | 1 | | | 2 | | | | | | 1 | | | 2 | | | | | | 2 | | | 2 | | | | | | 1 | | | 2 | | | | | | 1 | | | 2 | | | | | 2 | | | 2 | | | | | 2 | | | 2 | | | | | 2 | | | 1 | | | | | 1 | | | | 2 | | | | 1 | | | | 2 | | | | 1 | | | | 2 | | | | 2 | | | | 2 | | | | 2 | | | | 2 | | | | 1 | | | | 2 | | | | 1 | | | | 2 | | | | 2 | |
| 126 | | | 2 | | 2 | | | | | 2 | | | | | | 1 | | | | 2 | | | | | | 2 | | | | 2 | | | | | | 1 | | | | 2 | | | | | 2 | | | 1 | | | | | 1 | | | 2 | | | | | 2 | | | 1 | | | | | 1 | | | 1 | | | | | | 1 | | | 2 | | | | | | 1 | | | 2 | | | | | | 1 | | | 1 | | | | | | 1 | | | 2 | | | | | | 1 | | | 1 | | | | | | 1 | | | 1 | | | | | | 1 | | | 2 | | | | | | 2 | | | 1 | | | | | | 1 | | | 2 | | | | | | 1 | | | 2 | | | | | | 1 | | | 1 | | | | | 1 | | | 2 | | | | | 2 | | | 2 | | | | | 2 | | | 1 | | | | | 1 | | | | 2 | | | | 1 | | | | 2 | | | | 2 | | | | 2 | | | | 2 | | | | 2 | | | | 2 | | | | 2 | | | | 2 | | | | 2 | | | | 1 | | | | 2 | | | | 2 | |
| 127 | | | 2 | | 1 | | | | | 2 | | | | | | 2 | | | | 2 | | | | | | 1 | | | | 2 | | | | | | 1 | | | | 2 | | | | | 1 | | | 2 | | | | | 2 | | | 1 | | | | | 1 | | | 2 | | | | | 1 | | | 1 | | | | | | 1 | | | 2 | | | | | | 2 | | | 2 | | | | | | 1 | | | 1 | | | | | | 1 | | | 2 | | | | | | 2 | | | 1 | | | | | | 1 | | | 1 | | | | | | 1 | | | 2 | | | | | | 2 | | | 2 | | | | | | 1 | | | 2 | | | | | | 1 | | | 1 | | | | | | 1 | | | 2 | | | | | 1 | | | 2 | | | | | 2 | | | 2 | | | | | 1 | | | 2 | | | | | 1 | | | | 2 | | | | 2 | | | | 1 | | | | 1 | | | | 2 | | | | 1 | | | | 2 | | | | 1 | | | | 2 | | | | 1 | | | | 2 | | | | 2 | | | | 2 | | | | 2 | |
| 128 | | | 2 | | 1 | | | | | 2 | | | | | | 2 | | | | 2 | | | | | | 1 | | | | 2 | | | | | | 2 | | | | 2 | | | | | 1 | | | 2 | | | | | 1 | | | 1 | | | | | 1 | | | 1 | | | | | 1 | | | 2 | | | | | | 2 | | | 1 | | | | | | 1 | | | 2 | | | | | | 1 | | | 1 | | | | | | 1 | | | 2 | | | | | | 1 | | | 2 | | | | | | 2 | | | 2 | | | | | | 1 | | | 2 | | | | | | 1 | | | 2 | | | | | | 1 | | | 1 | | | | | | 1 | | | 1 | | | | | | 1 | | | 2 | | | | | 2 | | | 2 | | | | | 2 | | | 2 | | | | | 2 | | | 1 | | | | | 1 | | | | 2 | | | | 2 | | | | 2 | | | | 2 | | | | 2 | | | | 2 | | | | 2 | | | | 2 | | | | 2 | | | | 1 | | | | 2 | | | | 2 | | | | 2 | | | | 1 | |
| 129 | | | 2 | | 1 | | | | | 2 | | | | | | 2 | | | | 2 | | | | | | 1 | | | | 1 | | | | | | 1 | | | | 2 | | | | | 2 | | | 2 | | | | | 1 | | | 2 | | | | | 2 | | | 1 | | | | | 1 | | | 1 | | | | | | 1 | | | 2 | | | | | | 1 | | | 2 | | | | | | 2 | | | 1 | | | | | | 1 | | | 2 | | | | | | 1 | | | 2 | | | | | | 1 | | | 1 | | | | | | 1 | | | 2 | | | | | | 1 | | | 1 | | | | | | 1 | | | 2 | | | | | | 1 | | | 1 | | | | | | 1 | | | 2 | | | | | 2 | | | 2 | | | | | 1 | | | 2 | | | | | 2 | | | 1 | | | | | 1 | | | | 2 | | | | 1 | | | | 2 | | | | 1 | | | | 2 | | | | 1 | | | | 2 | | | | 1 | | | | 2 | | | | 1 | | | | 2 | | | | 1 | | | | 1 | | | | 1 | |
| 130 | | | 2 | | 2 | | | | | 2 | | | | | | 2 | | | | 2 | | | | | | 1 | | | | 2 | | | | | | 1 | | | | 2 | | | | | 2 | | | 2 | | | | | 1 | | | 2 | | | | | 1 | | | 1 | | | | | 1 | | | 2 | | | | | | 1 | | | 1 | | | | | | 1 | | | 2 | | | | | | 1 | | | 1 | | | | | | 1 | | | 1 | | | | | | 1 | | | 2 | | | | | | 1 | | | 1 | | | | | | 1 | | | 1 | | | | | | 1 | | | 1 | | | | | | 1 | | | 2 | | | | | | 1 | | | 2 | | | | | | 1 | | | 2 | | | | | 1 | | | 2 | | | | | 2 | | | 2 | | | | | 2 | | | 1 | | | | | 1 | | | | 2 | | | | 2 | | | | 2 | | | | 1 | | | | 2 | | | | 1 | | | | 2 | | | | 1 | | | | 2 | | | | 1 | | | | 2 | | | | 2 | | | | 2 | | | | 1 | |
| 131 | | | 1 | | 1 | | | | | 2 | | | | | | 2 | | | | 1 | | | | | | 1 | | | | 2 | | | | | | 1 | | | | 2 | | | | | 2 | | | 1 | | | | | 1 | | | 2 | | | | | 2 | | | 1 | | | | | 1 | | | 2 | | | | | | 2 | | | 2 | | | | | | 1 | | | 2 | | | | | | 1 | | | 1 | | | | | | 1 | | | 2 | | | | | | 1 | | | 2 | | | | | | 2 | | | 2 | | | | | | 2 | | | 2 | | | | | | 2 | | | 1 | | | | | | 1 | | | 2 | | | | | | 2 | | | 1 | | | | | | 1 | | | 2 | | | | | 1 | | | 2 | | | | | 2 | | | 2 | | | | | 2 | | | 1 | | | | | 1 | | | | 2 | | | | 2 | | | | 2 | | | | 1 | | | | 1 | | | | 1 | | | | 2 | | | | 2 | | | | 2 | | | | 1 | | | | 2 | | | | 1 | | | | 2 | | | | 1 | |
| 132 | | | 2 | | 1 | | | | | 2 | | | | | | 2 | | | | 1 | | | | | | 1 | | | | 1 | | | | | | 1 | | | | 2 | | | | | 2 | | | 2 | | | | | 1 | | | 2 | | | | | 1 | | | 1 | | | | | 1 | | | 2 | | | | | | 1 | | | 2 | | | | | | 1 | | | 2 | | | | | | 1 | | | 1 | | | | | | 1 | | | 2 | | | | | | 2 | | | 1 | | | | | | 1 | | | 2 | | | | | | 2 | | | 2 | | | | | | 2 | | | 2 | | | | | | 2 | | | 2 | | | | | | 2 | | | 1 | | | | | | 1 | | | 2 | | | | | 2 | | | 2 | | | | | 2 | | | 2 | | | | | 2 | | | 1 | | | | | 1 | | | | 2 | | | | 2 | | | | 2 | | | | 1 | | | | 2 | | | | 1 | | | | 2 | | | | 2 | | | | 2 | | | | 2 | | | | 2 | | | | 1 | | | | 1 | | | | 1 | |
| 133 | | | 2 | | 2 | | | | | 2 | | | | | | 2 | | | | 2 | | | | | | 1 | | | | 2 | | | | | | 2 | | | | 2 | | | | | 2 | | | 2 | | | | | 2 | | | 2 | | | | | 1 | | | 1 | | | | | 1 | | | 1 | | | | | | 1 | | | 1 | | | | | | 1 | | | 2 | | | | | | 2 | | | 1 | | | | | | 1 | | | 2 | | | | | | 2 | | | 2 | | | | | | 1 | | | 2 | | | | | | 1 | | | 2 | | | | | | 2 | | | 2 | | | | | | 2 | | | 2 | | | | | | 2 | | | 1 | | | | | | 1 | | | 2 | | | | | 1 | | | 2 | | | | | 2 | | | 2 | | | | | 2 | | | 2 | | | | | 1 | | | | 2 | | | | 2 | | | | 2 | | | | 2 | | | | 2 | | | | 1 | | | | 2 | | | | 1 | | | | 2 | | | | 2 | | | | 1 | | | | 1 | | | | 1 | | | | 1 | |
| 134 | | | 2 | | 2 | | | | | 2 | | | | | | 2 | | | | 2 | | | | | | 1 | | | | 2 | | | | | | 2 | | | | 2 | | | | | 2 | | | 2 | | | | | 2 | | | 2 | | | | | 1 | | | 1 | | | | | 1 | | | 1 | | | | | | 1 | | | 1 | | | | | | 1 | | | 2 | | | | | | 2 | | | 1 | | | | | | 1 | | | 2 | | | | | | 2 | | | 2 | | | | | | 1 | | | 2 | | | | | | 1 | | | 2 | | | | | | 2 | | | 2 | | | | | | 2 | | | 2 | | | | | | 2 | | | 1 | | | | | | 1 | | | 2 | | | | | 1 | | | 2 | | | | | 2 | | | 2 | | | | | 2 | | | 1 | | | | | 1 | | | | 2 | | | | 2 | | | | 2 | | | | 2 | | | | 2 | | | | 1 | | | | 2 | | | | 1 | | | | 2 | | | | 2 | | | | 1 | | | | 1 | | | | 1 | | | | 1 | |
| 135 | | | 2 | | 1 | | | | | 2 | | | | | | 2 | | | | 2 | | | | | | 1 | | | | 2 | | | | | | 1 | | | | 2 | | | | | 2 | | | 2 | | | | | 2 | | | 1 | | | | | 1 | | | 1 | | | | | 1 | | | 2 | | | | | | 1 | | | 2 | | | | | | 1 | | | 2 | | | | | | 2 | | | 1 | | | | | | 1 | | | 2 | | | | | | 1 | | | 1 | | | | | | 1 | | | 2 | | | | | | 1 | | | 2 | | | | | | 1 | | | 2 | | | | | | 1 | | | 2 | | | | | | 1 | | | 2 | | | | | | 1 | | | 2 | | | | | 1 | | | 2 | | | | | 1 | | | 2 | | | | | 2 | | | 2 | | | | | 1 | | | | 2 | | | | 2 | | | | 2 | | | | 1 | | | | 1 | | | | 1 | | | | 2 | | | | 2 | | | | 2 | | | | 1 | | | | 2 | | | | 1 | | | | 2 | | | | 2 | |
| 136 | | | 1 | | 1 | | | | | 2 | | | | | | 2 | | | | 1 | | | | | | 1 | | | | 1 | | | | | | 1 | | | | 2 | | | | | 1 | | | 1 | | | | | 1 | | | 1 | | | | | 1 | | | 1 | | | | | 1 | | | 1 | | | | | | 1 | | | 2 | | | | | | 1 | | | 2 | | | | | | 2 | | | 1 | | | | | | 1 | | | 2 | | | | | | 2 | | | 1 | | | | | | 1 | | | 1 | | | | | | 1 | | | 1 | | | | | | 1 | | | 2 | | | | | | 1 | | | 2 | | | | | | 1 | | | 1 | | | | | | 1 | | | 1 | | | | | 1 | | | 2 | | | | | 2 | | | 2 | | | | | 2 | | | 1 | | | | | 1 | | | | 2 | | | | 1 | | | | 1 | | | | 1 | | | | 2 | | | | 2 | | | | 2 | | | | 2 | | | | 1 | | | | 1 | | | | 2 | | | | 1 | | | | 2 | | | | 1 | |
| 137 | | | 2 | | 1 | | | | | 1 | | | | | | 1 | | | | 2 | | | | | | 2 | | | | 2 | | | | | | 1 | | | | 2 | | | | | 1 | | | 2 | | | | | 2 | | | 2 | | | | | 1 | | | 1 | | | | | 1 | | | 2 | | | | | | 1 | | | 2 | | | | | | 1 | | | 2 | | | | | | 1 | | | 2 | | | | | | 1 | | | 2 | | | | | | 1 | | | 1 | | | | | | 1 | | | 2 | | | | | | 2 | | | 2 | | | | | | 2 | | | 2 | | | | | | 1 | | | 2 | | | | | | 2 | | | 2 | | | | | | 1 | | | 2 | | | | | 2 | | | 2 | | | | | 2 | | | 2 | | | | | 1 | | | 1 | | | | | 1 | | | | 2 | | | | 2 | | | | 2 | | | | 2 | | | | 2 | | | | 2 | | | | 2 | | | | 2 | | | | 2 | | | | 1 | | | | 2 | | | | 1 | | | | 2 | | | | 1 | |
| 138 | | | 2 | | 1 | | | | | 2 | | | | | | 1 | | | | 2 | | | | | | 1 | | | | 2 | | | | | | 2 | | | | 2 | | | | | 2 | | | 2 | | | | | 1 | | | 1 | | | | | 1 | | | 1 | | | | | 1 | | | 2 | | | | | | 1 | | | 2 | | | | | | 1 | | | 1 | | | | | | 1 | | | 1 | | | | | | 1 | | | 2 | | | | | | 2 | | | 1 | | | | | | 1 | | | 1 | | | | | | 1 | | | 2 | | | | | | 2 | | | 1 | | | | | | 1 | | | 2 | | | | | | 2 | | | 1 | | | | | | 1 | | | 2 | | | | | 2 | | | 2 | | | | | 1 | | | 2 | | | | | 2 | | | 2 | | | | | 1 | | | | 2 | | | | 1 | | | | 2 | | | | 1 | | | | 2 | | | | 1 | | | | 2 | | | | 1 | | | | 2 | | | | 1 | | | | 2 | | | | 1 | | | | 2 | | | | 1 | |
| 139 | | | 2 | | 2 | | | | | 2 | | | | | | 2 | | | | 1 | | | | | | 1 | | | | 2 | | | | | | 2 | | | | 2 | | | | | 2 | | | 1 | | | | | 1 | | | 2 | | | | | 1 | | | 1 | | | | | 1 | | | 1 | | | | | | 1 | | | 2 | | | | | | 1 | | | 2 | | | | | | 1 | | | 1 | | | | | | 1 | | | 2 | | | | | | 2 | | | 1 | | | | | | 1 | | | 2 | | | | | | 2 | | | 2 | | | | | | 1 | | | 2 | | | | | | 2 | | | 2 | | | | | | 2 | | | 1 | | | | | | 1 | | | 1 | | | | | 1 | | | 2 | | | | | 2 | | | 2 | | | | | 2 | | | 1 | | | | | 1 | | | | 2 | | | | 1 | | | | 2 | | | | 1 | | | | 2 | | | | 2 | | | | 2 | | | | 2 | | | | 2 | | | | 1 | | | | 2 | | | | 1 | | | | 1 | | | | 1 | |
| 141 | | | 1 | | 1 | | | | | 2 | | | | | | 2 | | | | 2 | | | | | | 2 | | | | 2 | | | | | | 1 | | | | 2 | | | | | 1 | | | 1 | | | | | 1 | | | 2 | | | | | 2 | | | 1 | | | | | 1 | | | 2 | | | | | | 1 | | | 1 | | | | | | 1 | | | 2 | | | | | | 1 | | | 1 | | | | | | 1 | | | 2 | | | | | | 2 | | | 2 | | | | | | 1 | | | 1 | | | | | | 1 | | | 2 | | | | | | 1 | | | 2 | | | | | | 1 | | | 2 | | | | | | 1 | | | 2 | | | | | | 1 | | | 2 | | | | | 1 | | | 2 | | | | | 2 | | | 2 | | | | | 1 | | | 1 | | | | | 1 | | | | 2 | | | | 1 | | | | 2 | | | | 1 | | | | 2 | | | | 1 | | | | 2 | | | | 2 | | | | 2 | | | | 2 | | | | 2 | | | | 1 | | | | 2 | | | | 2 | |
| 142 | | | 2 | | 1 | | | | | 2 | | | | | | 1 | | | | 1 | | | | | | 1 | | | | 1 | | | | | | 1 | | | | 2 | | | | | 1 | | | 1 | | | | | 1 | | | 2 | | | | | 2 | | | 1 | | | | | 1 | | | 1 | | | | | | 1 | | | 1 | | | | | | 1 | | | 2 | | | | | | 1 | | | 1 | | | | | | 1 | | | 2 | | | | | | 1 | | | 2 | | | | | | 1 | | | 2 | | | | | | 1 | | | 2 | | | | | | 2 | | | 1 | | | | | | 1 | | | 1 | | | | | | 1 | | | 2 | | | | | | 1 | | | 2 | | | | | 1 | | | 2 | | | | | 2 | | | 2 | | | | | 2 | | | 1 | | | | | 1 | | | | 2 | | | | 2 | | | | 2 | | | | 2 | | | | 2 | | | | 1 | | | | 2 | | | | 2 | | | | 2 | | | | 1 | | | | 2 | | | | 2 | | | | 1 | | | | 1 | |
| Sample ID | | HLD  6 | | | | | | | HLD  *Supplementary Table (Continued)*  39 | | | | | | | | | | | | | HLD  40 | | | | | | | | | | HLD  45 | | | | | | | | | | HLD  48 | | | | | | | | HLD  56 | | | | | | | | HLD  58 | | | | | | | | HLD  64 | | | | | | | | | HLD  67 | | | | | | | | | HLD  70 | | | | | | | | | HLD  77 | | | | | | | | | HLD  81 | | | | | | | | | HLD  83 | | | | | | | | | HLD  84 | | | | | | | | | HLD  88 | | | | | | | | | HLD  92 | | | | | | | | | HLD  93 | | | | | | | | | HLD  97 | | | | | | | | | HLD  99 | | | | | | | | HLD  101 | | | | | | | | HLD  111 | | | | | | | | HLD  114 | | | | | | | | HLD  118 | | | | | | | | HLD  122 | | | | | | | | HLD  124 | | | | | | | | HLD  125 | | | | | | | | HLD  128 | | | | | | | | HLD  131 | | | | | | | | HLD  133 | | | | | | | | HLD  136 | | | | |
| 143 | | | | 2 | | 1 | | 2 | | | | | | 1 | | | | 2 | | | | | | 1 | | | | 1 | | | | | | 1 | | | | 1 | | | | | | 1 | | | | 1 | | | | | 1 | | | 2 | | | | | 1 | | | 1 | | | | | 1 | | | 1 | | | | | | 1 | | | 2 | | | | | | 1 | | | 2 | | | | | | 2 | | | 1 | | | | | | 1 | | | 2 | | | | | | 1 | | | 2 | | | | | | 1 | | | 2 | | | | | | 1 | | | 2 | | | | | | 1 | | | 1 | | | | | | 1 | | | 2 | | | | | | 1 | | | 1 | | | | | | 1 | | | 2 | | | | | 1 | | | 2 | | | | | 2 | | | 1 | | | | | 1 | | | 1 | | | | | 1 | | | | 2 | | | | 2 | | | | 2 | | | | 1 | | | | 2 | | | | 1 | | | | 2 | | | | 2 | | | | 2 | | | | 1 | | | | 1 | | | | 1 | | | | 1 | | | | 1 | |
| 144 | | | | 1 | | 1 | | 2 | | | | | | 2 | | | | 1 | | | | | | 1 | | | | 2 | | | | | | 1 | | | | 1 | | | | | | 1 | | | | 2 | | | | | 1 | | | 2 | | | | | 1 | | | 1 | | | | | 1 | | | 2 | | | | | | 1 | | | 2 | | | | | | 1 | | | 2 | | | | | | 2 | | | 1 | | | | | | 1 | | | 2 | | | | | | 1 | | | 2 | | | | | | 1 | | | 1 | | | | | | 1 | | | 1 | | | | | | 1 | | | 2 | | | | | | 1 | | | 2 | | | | | | 2 | | | 1 | | | | | | 1 | | | 2 | | | | | 1 | | | 1 | | | | | 1 | | | 2 | | | | | 1 | | | 1 | | | | | 1 | | | | 2 | | | | 1 | | | | 2 | | | | 1 | | | | 2 | | | | 1 | | | | 2 | | | | 2 | | | | 1 | | | | 1 | | | | 1 | | | | 1 | | | | 1 | | | | 1 | |
| 145 | | | | 2 | | 1 | | 2 | | | | | | 1 | | | | 1 | | | | | | 1 | | | | 1 | | | | | | 1 | | | | 2 | | | | | | 2 | | | | 1 | | | | | 1 | | | 2 | | | | | 2 | | | 2 | | | | | 1 | | | 1 | | | | | | 1 | | | 2 | | | | | | 2 | | | 2 | | | | | | 2 | | | 1 | | | | | | 1 | | | 2 | | | | | | 2 | | | 1 | | | | | | 1 | | | 2 | | | | | | 1 | | | 2 | | | | | | 1 | | | 1 | | | | | | 1 | | | 2 | | | | | | 2 | | | 1 | | | | | | 1 | | | 1 | | | | | 1 | | | 2 | | | | | 2 | | | 2 | | | | | 1 | | | 1 | | | | | 1 | | | | 2 | | | | 2 | | | | 1 | | | | 1 | | | | 2 | | | | 1 | | | | 2 | | | | 1 | | | | 2 | | | | 1 | | | | 2 | | | | 1 | | | | 2 | | | | 2 | |
| 147 | | | | 2 | | 1 | | 2 | | | | | | 2 | | | | 2 | | | | | | 2 | | | | 2 | | | | | | 1 | | | | 2 | | | | | | 2 | | | | 2 | | | | | 1 | | | 1 | | | | | 1 | | | 1 | | | | | 1 | | | 2 | | | | | | 1 | | | 2 | | | | | | 1 | | | 2 | | | | | | 2 | | | 1 | | | | | | 1 | | | 1 | | | | | | 1 | | | 2 | | | | | | 1 | | | 2 | | | | | | 1 | | | 2 | | | | | | 1 | | | 2 | | | | | | 1 | | | 1 | | | | | | 1 | | | 1 | | | | | | 1 | | | 2 | | | | | 2 | | | 2 | | | | | 2 | | | 2 | | | | | 2 | | | 1 | | | | | 1 | | | | 2 | | | | 2 | | | | 1 | | | | 1 | | | | 2 | | | | 2 | | | | 2 | | | | 2 | | | | 1 | | | | 1 | | | | 2 | | | | 1 | | | | 2 | | | | 2 | |
| 148 | | | | 2 | | 2 | | 2 | | | | | | 1 | | | | 2 | | | | | | 1 | | | | 1 | | | | | | 1 | | | | 1 | | | | | | 1 | | | | 1 | | | | | 1 | | | 2 | | | | | 2 | | | 1 | | | | | 1 | | | 2 | | | | | | 1 | | | 2 | | | | | | 1 | | | 2 | | | | | | 1 | | | 1 | | | | | | 1 | | | 2 | | | | | | 1 | | | 1 | | | | | | 1 | | | 2 | | | | | | 1 | | | 1 | | | | | | 1 | | | 2 | | | | | | 1 | | | 2 | | | | | | 2 | | | 2 | | | | | | 1 | | | 2 | | | | | 2 | | | 2 | | | | | 1 | | | 2 | | | | | 2 | | | 1 | | | | | 1 | | | | 2 | | | | 1 | | | | 1 | | | | 1 | | | | 2 | | | | 1 | | | | 2 | | | | 2 | | | | 1 | | | | 1 | | | | 2 | | | | 2 | | | | 2 | | | | 2 | |
| 149 | | | | 2 | | 2 | | 2 | | | | | | 2 | | | | 2 | | | | | | 1 | | | | 1 | | | | | | 1 | | | | 2 | | | | | | 1 | | | | 1 | | | | | 1 | | | 2 | | | | | 2 | | | 2 | | | | | 1 | | | 2 | | | | | | 1 | | | 2 | | | | | | 1 | | | 2 | | | | | | 1 | | | 1 | | | | | | 1 | | | 2 | | | | | | 2 | | | 1 | | | | | | 1 | | | 2 | | | | | | 1 | | | 1 | | | | | | 1 | | | 2 | | | | | | 1 | | | 2 | | | | | | 2 | | | 1 | | | | | | 1 | | | 2 | | | | | 1 | | | 2 | | | | | 2 | | | 2 | | | | | 2 | | | 1 | | | | | 1 | | | | 2 | | | | 2 | | | | 1 | | | | 1 | | | | 2 | | | | 1 | | | | 2 | | | | 1 | | | | 2 | | | | 2 | | | | 2 | | | | 1 | | | | 1 | | | | 1 | |
| 150 | | | | 2 | | 1 | | 2 | | | | | | 2 | | | | 2 | | | | | | 1 | | | | 1 | | | | | | 1 | | | | 2 | | | | | | 1 | | | | 1 | | | | | 1 | | | 2 | | | | | 2 | | | 1 | | | | | 1 | | | 1 | | | | | | 1 | | | 1 | | | | | | 1 | | | 2 | | | | | | 2 | | | 1 | | | | | | 1 | | | 2 | | | | | | 1 | | | 1 | | | | | | 1 | | | 2 | | | | | | 1 | | | 1 | | | | | | 1 | | | 2 | | | | | | 2 | | | 2 | | | | | | 2 | | | 1 | | | | | | 1 | | | 1 | | | | | 1 | | | 2 | | | | | 1 | | | 2 | | | | | 2 | | | 1 | | | | | 1 | | | | 2 | | | | 2 | | | | 2 | | | | 1 | | | | 1 | | | | 1 | | | | 2 | | | | 2 | | | | 2 | | | | 2 | | | | 2 | | | | 1 | | | | 2 | | | | 2 | |
| 151 | | | | 1 | | 1 | | 2 | | | | | | 2 | | | | 2 | | | | | | 2 | | | | 2 | | | | | | 1 | | | | 2 | | | | | | 1 | | | | 2 | | | | | 2 | | | 2 | | | | | 1 | | | 1 | | | | | 1 | | | 2 | | | | | | 1 | | | 2 | | | | | | 1 | | | 2 | | | | | | 1 | | | 1 | | | | | | 1 | | | 2 | | | | | | 2 | | | 1 | | | | | | 1 | | | 2 | | | | | | 2 | | | 2 | | | | | | 1 | | | 2 | | | | | | 1 | | | 2 | | | | | | 1 | | | 1 | | | | | | 1 | | | 2 | | | | | 2 | | | 2 | | | | | 2 | | | 2 | | | | | 2 | | | 1 | | | | | 1 | | | | 1 | | | | 1 | | | | 1 | | | | 1 | | | | 2 | | | | 1 | | | | 2 | | | | 2 | | | | 2 | | | | 1 | | | | 2 | | | | 2 | | | | 1 | | | | 1 | |
| 152 | | | | 2 | | 2 | | 2 | | | | | | 2 | | | | 2 | | | | | | 1 | | | | 2 | | | | | | 2 | | | | 1 | | | | | | 1 | | | | 2 | | | | | 1 | | | 2 | | | | | 1 | | | 1 | | | | | 1 | | | 2 | | | | | | 2 | | | 1 | | | | | | 1 | | | 2 | | | | | | 1 | | | 1 | | | | | | 1 | | | 2 | | | | | | 1 | | | 1 | | | | | | 1 | | | 2 | | | | | | 1 | | | 1 | | | | | | 1 | | | 1 | | | | | | 1 | | | 2 | | | | | | 1 | | | 2 | | | | | | 1 | | | 2 | | | | | 1 | | | 2 | | | | | 2 | | | 2 | | | | | 2 | | | 1 | | | | | 1 | | | | 2 | | | | 2 | | | | 2 | | | | 1 | | | | 2 | | | | 1 | | | | 2 | | | | 2 | | | | 1 | | | | 1 | | | | 2 | | | | 1 | | | | 2 | | | | 1 | |
| 153 | | | | 2 | | 2 | | 2 | | | | | | 2 | | | | 1 | | | | | | 1 | | | | 1 | | | | | | 1 | | | | 1 | | | | | | 1 | | | | 2 | | | | | 2 | | | 2 | | | | | 2 | | | 1 | | | | | 1 | | | 1 | | | | | | 1 | | | 2 | | | | | | 1 | | | 2 | | | | | | 1 | | | 1 | | | | | | 1 | | | 2 | | | | | | 1 | | | 1 | | | | | | 1 | | | 1 | | | | | | 1 | | | 2 | | | | | | 1 | | | 1 | | | | | | 1 | | | 1 | | | | | | 1 | | | 1 | | | | | | 1 | | | 2 | | | | | 1 | | | 2 | | | | | 2 | | | 2 | | | | | 2 | | | 1 | | | | | 1 | | | | 2 | | | | 2 | | | | 2 | | | | 1 | | | | 2 | | | | 2 | | | | 1 | | | | 1 | | | | 2 | | | | 2 | | | | 2 | | | | 1 | | | | 2 | | | | 1 | |
| 155 | | | | 2 | | 2 | | 2 | | | | | | 2 | | | | 2 | | | | | | 2 | | | | 2 | | | | | | 1 | | | | 2 | | | | | | 1 | | | | 2 | | | | | 1 | | | 2 | | | | | 1 | | | 2 | | | | | 1 | | | 2 | | | | | | 1 | | | 1 | | | | | | 1 | | | 2 | | | | | | 1 | | | 1 | | | | | | 1 | | | 2 | | | | | | 2 | | | 1 | | | | | | 1 | | | 2 | | | | | | 2 | | | 2 | | | | | | 2 | | | 2 | | | | | | 1 | | | 1 | | | | | | 1 | | | 1 | | | | | | 1 | | | 2 | | | | | 1 | | | 2 | | | | | 2 | | | 2 | | | | | 1 | | | 1 | | | | | 1 | | | | 2 | | | | 2 | | | | 2 | | | | 2 | | | | 1 | | | | 1 | | | | 2 | | | | 1 | | | | 2 | | | | 1 | | | | 2 | | | | 1 | | | | 2 | | | | 1 | |
| 156 | | | | 2 | | 2 | | 2 | | | | | | 1 | | | | 2 | | | | | | 1 | | | | 1 | | | | | | 1 | | | | 2 | | | | | | 2 | | | | 1 | | | | | 1 | | | 2 | | | | | 2 | | | 1 | | | | | 1 | | | 1 | | | | | | 1 | | | 2 | | | | | | 2 | | | 2 | | | | | | 1 | | | 1 | | | | | | 1 | | | 2 | | | | | | 1 | | | 1 | | | | | | 1 | | | 1 | | | | | | 1 | | | 2 | | | | | | 2 | | | 1 | | | | | | 1 | | | 2 | | | | | | 2 | | | 2 | | | | | | 2 | | | 2 | | | | | 1 | | | 2 | | | | | 2 | | | 2 | | | | | 2 | | | 1 | | | | | 1 | | | | 2 | | | | 2 | | | | 2 | | | | 1 | | | | 1 | | | | 1 | | | | 2 | | | | 1 | | | | 2 | | | | 1 | | | | 2 | | | | 2 | | | | 1 | | | | 1 | |
| 157 | | | | 2 | | 1 | | 2 | | | | | | 2 | | | | 1 | | | | | | 1 | | | | 2 | | | | | | 1 | | | | 2 | | | | | | 1 | | | | 2 | | | | | 2 | | | 2 | | | | | 1 | | | 1 | | | | | 1 | | | 2 | | | | | | 1 | | | 1 | | | | | | 1 | | | 1 | | | | | | 1 | | | 1 | | | | | | 1 | | | 2 | | | | | | 2 | | | 2 | | | | | | 2 | | | 2 | | | | | | 1 | | | 2 | | | | | | 2 | | | 1 | | | | | | 1 | | | 2 | | | | | | 1 | | | 1 | | | | | | 1 | | | 2 | | | | | 1 | | | 2 | | | | | 2 | | | 2 | | | | | 1 | | | 1 | | | | | 1 | | | | 2 | | | | 1 | | | | 2 | | | | 2 | | | | 2 | | | | 1 | | | | 2 | | | | 2 | | | | 2 | | | | 1 | | | | 2 | | | | 1 | | | | 1 | | | | 1 | |
| 158 | | | | 2 | | 2 | | 2 | | | | | | 2 | | | | 1 | | | | | | 1 | | | | 2 | | | | | | 1 | | | | 2 | | | | | | 2 | | | | 1 | | | | | 1 | | | 2 | | | | | 1 | | | 1 | | | | | 1 | | | 2 | | | | | | 1 | | | 2 | | | | | | 1 | | | 2 | | | | | | 2 | | | 2 | | | | | | 2 | | | 2 | | | | | | 1 | | | 1 | | | | | | 1 | | | 1 | | | | | | 1 | | | 2 | | | | | | 1 | | | 2 | | | | | | 2 | | | 2 | | | | | | 2 | | | 2 | | | | | | 1 | | | 2 | | | | | 1 | | | 2 | | | | | 2 | | | 2 | | | | | 1 | | | 1 | | | | | 1 | | | | 2 | | | | 1 | | | | 2 | | | | 1 | | | | 2 | | | | 1 | | | | 2 | | | | 2 | | | | 2 | | | | 2 | | | | 2 | | | | 1 | | | | 1 | | | | 1 | |
| 159 | | | | 2 | | 1 | | 2 | | | | | | 2 | | | | 2 | | | | | | 1 | | | | 2 | | | | | | 2 | | | | 2 | | | | | | 1 | | | | 2 | | | | | 1 | | | 2 | | | | | 1 | | | 1 | | | | | 1 | | | 2 | | | | | | 1 | | | 2 | | | | | | 2 | | | 1 | | | | | | 1 | | | 1 | | | | | | 1 | | | 2 | | | | | | 2 | | | 1 | | | | | | 1 | | | 2 | | | | | | 1 | | | 2 | | | | | | 1 | | | 1 | | | | | | 1 | | | 2 | | | | | | 2 | | | 1 | | | | | | 1 | | | 2 | | | | | 2 | | | 2 | | | | | 2 | | | 2 | | | | | 2 | | | 2 | | | | | 1 | | | | 2 | | | | 2 | | | | 2 | | | | 2 | | | | 2 | | | | 2 | | | | 2 | | | | 1 | | | | 2 | | | | 1 | | | | 2 | | | | 1 | | | | 2 | | | | 2 | |
| 160 | | | | 1 | | 1 | | 2 | | | | | | 1 | | | | 2 | | | | | | 2 | | | | 1 | | | | | | 1 | | | | 1 | | | | | | 1 | | | | 2 | | | | | 2 | | | 2 | | | | | 2 | | | 1 | | | | | 1 | | | 1 | | | | | | 1 | | | 2 | | | | | | 2 | | | 2 | | | | | | 2 | | | 1 | | | | | | 1 | | | 1 | | | | | | 1 | | | 1 | | | | | | 1 | | | 2 | | | | | | 2 | | | 1 | | | | | | 1 | | | 1 | | | | | | 1 | | | 1 | | | | | | 1 | | | 1 | | | | | | 1 | | | 2 | | | | | 1 | | | 2 | | | | | 1 | | | 2 | | | | | 2 | | | 1 | | | | | 1 | | | | 2 | | | | 2 | | | | 2 | | | | 2 | | | | 2 | | | | 2 | | | | 2 | | | | 2 | | | | 2 | | | | 1 | | | | 1 | | | | 1 | | | | 2 | | | | 1 | |
| 161 | | | | 2 | | 2 | | 2 | | | | | | 1 | | | | 1 | | | | | | 1 | | | | 2 | | | | | | 1 | | | | 2 | | | | | | 2 | | | | 1 | | | | | 1 | | | 2 | | | | | 1 | | | 2 | | | | | 1 | | | 2 | | | | | | 1 | | | 1 | | | | | | 1 | | | 2 | | | | | | 1 | | | 2 | | | | | | 1 | | | 2 | | | | | | 2 | | | 2 | | | | | | 1 | | | 2 | | | | | | 2 | | | 1 | | | | | | 1 | | | 1 | | | | | | 1 | | | 2 | | | | | | 2 | | | 1 | | | | | | 1 | | | 1 | | | | | 1 | | | 2 | | | | | 2 | | | 1 | | | | | 1 | | | 1 | | | | | 1 | | | | 2 | | | | 1 | | | | 2 | | | | 2 | | | | 1 | | | | 1 | | | | 2 | | | | 1 | | | | 1 | | | | 1 | | | | 2 | | | | 2 | | | | 1 | | | | 1 | |
| 162 | | | | 1 | | 1 | | 2 | | | | | | 2 | | | | 1 | | | | | | 1 | | | | 2 | | | | | | 1 | | | | 2 | | | | | | 2 | | | | 2 | | | | | 1 | | | 2 | | | | | 2 | | | 1 | | | | | 1 | | | 1 | | | | | | 1 | | | 1 | | | | | | 1 | | | 2 | | | | | | 1 | | | 1 | | | | | | 1 | | | 2 | | | | | | 2 | | | 1 | | | | | | 1 | | | 2 | | | | | | 1 | | | 2 | | | | | | 1 | | | 2 | | | | | | 1 | | | 2 | | | | | | 1 | | | 2 | | | | | | 1 | | | 2 | | | | | 1 | | | 2 | | | | | 2 | | | 2 | | | | | 2 | | | 1 | | | | | 1 | | | | 2 | | | | 2 | | | | 2 | | | | 2 | | | | 2 | | | | 1 | | | | 2 | | | | 1 | | | | 2 | | | | 1 | | | | 1 | | | | 1 | | | | 2 | | | | 2 | |
| 163 | | | | 1 | | 1 | | 2 | | | | | | 2 | | | | 2 | | | | | | 2 | | | | 1 | | | | | | 1 | | | | 2 | | | | | | 2 | | | | 1 | | | | | 1 | | | 2 | | | | | 2 | | | 1 | | | | | 1 | | | 2 | | | | | | 1 | | | 1 | | | | | | 1 | | | 2 | | | | | | 1 | | | 1 | | | | | | 1 | | | 2 | | | | | | 2 | | | 2 | | | | | | 1 | | | 2 | | | | | | 1 | | | 2 | | | | | | 1 | | | 2 | | | | | | 1 | | | 2 | | | | | | 1 | | | 2 | | | | | | 1 | | | 2 | | | | | 1 | | | 2 | | | | | 2 | | | 2 | | | | | 1 | | | 1 | | | | | 1 | | | | 2 | | | | 1 | | | | 2 | | | | 2 | | | | 2 | | | | 1 | | | | 2 | | | | 2 | | | | 2 | | | | 1 | | | | 1 | | | | 1 | | | | 2 | | | | 1 | |
| 164 | | | | 2 | | 1 | | 2 | | | | | | 1 | | | | 2 | | | | | | 2 | | | | 2 | | | | | | 1 | | | | 2 | | | | | | 2 | | | | 2 | | | | | 2 | | | 2 | | | | | 1 | | | 1 | | | | | 1 | | | 1 | | | | | | 1 | | | 2 | | | | | | 1 | | | 2 | | | | | | 1 | | | 1 | | | | | | 1 | | | 2 | | | | | | 1 | | | 2 | | | | | | 1 | | | 2 | | | | | | 1 | | | 2 | | | | | | 1 | | | 1 | | | | | | 1 | | | 2 | | | | | | 1 | | | 2 | | | | | | 1 | | | 1 | | | | | 1 | | | 2 | | | | | 2 | | | 2 | | | | | 2 | | | 1 | | | | | 1 | | | | 2 | | | | 2 | | | | 2 | | | | 2 | | | | 2 | | | | 1 | | | | 2 | | | | 1 | | | | 2 | | | | 1 | | | | 2 | | | | 2 | | | | 2 | | | | 2 | |
| 165 | | | | 2 | | 1 | | 2 | | | | | | 2 | | | | 2 | | | | | | 2 | | | | 2 | | | | | | 1 | | | | 2 | | | | | | 1 | | | | 1 | | | | | 1 | | | 2 | | | | | 1 | | | 1 | | | | | 1 | | | 1 | | | | | | 1 | | | 2 | | | | | | 2 | | | 2 | | | | | | 1 | | | 1 | | | | | | 1 | | | 2 | | | | | | 1 | | | 1 | | | | | | 1 | | | 1 | | | | | | 1 | | | 2 | | | | | | 1 | | | 2 | | | | | | 1 | | | 2 | | | | | | 2 | | | 1 | | | | | | 1 | | | 2 | | | | | 1 | | | 2 | | | | | 2 | | | 2 | | | | | 2 | | | 1 | | | | | 1 | | | | 2 | | | | 1 | | | | 2 | | | | 1 | | | | 2 | | | | 2 | | | | 2 | | | | 2 | | | | 1 | | | | 1 | | | | 2 | | | | 1 | | | | 2 | | | | 1 | |
| 166 | | | | 1 | | 1 | | 2 | | | | | | 2 | | | | 1 | | | | | | 1 | | | | 1 | | | | | | 1 | | | | 2 | | | | | | 1 | | | | 1 | | | | | 1 | | | 2 | | | | | 1 | | | 1 | | | | | 1 | | | 1 | | | | | | 1 | | | 1 | | | | | | 1 | | | 2 | | | | | | 2 | | | 1 | | | | | | 1 | | | 2 | | | | | | 2 | | | 1 | | | | | | 1 | | | 2 | | | | | | 2 | | | 1 | | | | | | 1 | | | 2 | | | | | | 1 | | | 2 | | | | | | 1 | | | 1 | | | | | | 1 | | | 2 | | | | | 2 | | | 2 | | | | | 2 | | | 2 | | | | | 1 | | | 1 | | | | | 1 | | | | 2 | | | | 2 | | | | 1 | | | | 1 | | | | 2 | | | | 2 | | | | 2 | | | | 1 | | | | 2 | | | | 1 | | | | 1 | | | | 1 | | | | 2 | | | | 1 | |
| Sample ID | | HLD  *Supplementary Table (Continued)*  6 | | | | | | | HLD  39 | | | | | | | | | | | | | HLD  40 | | | | | | | | | | HLD  45 | | | | | | | | | | HLD  48 | | | | | | | | HLD  56 | | | | | | | | HLD  58 | | | | | | | | HLD  64 | | | | | | | | | HLD  67 | | | | | | | | | HLD  70 | | | | | | | | | HLD  77 | | | | | | | | | HLD  81 | | | | | | | | | HLD  83 | | | | | | | | | HLD  84 | | | | | | | | | HLD  88 | | | | | | | | | HLD  92 | | | | | | | | | HLD  93 | | | | | | | | | HLD  97 | | | | | | | | | HLD  99 | | | | | | | | HLD  101 | | | | | | | | HLD  111 | | | | | | | | HLD  114 | | | | | | | | HLD  118 | | | | | | | | HLD  122 | | | | | | | | HLD  124 | | | | | | | | HLD  125 | | | | | | | | HLD  128 | | | | | | | | HLD  131 | | | | | | | | HLD  133 | | | | | | | | HLD  136 | | | | |
| 167 | | | | 2 | | 2 | | 2 | | | | | | 2 | | | | 1 | | | | | | 1 | | | | 2 | | | | | | 2 | | | | 2 | | | | | | 2 | | | | 2 | | | | | 1 | | | 2 | | | | | 2 | | | 1 | | | | | 1 | | | 2 | | | | | | 2 | | | 1 | | | | | | 1 | | | 2 | | | | | | 1 | | | 1 | | | | | | 1 | | | 2 | | | | | | 2 | | | 1 | | | | | | 1 | | | 2 | | | | | | 1 | | | 2 | | | | | | 1 | | | 1 | | | | | | 1 | | | 2 | | | | | | 2 | | | 2 | | | | | | 1 | | | 2 | | | | | 2 | | | 2 | | | | | 2 | | | 2 | | | | | 2 | | | 1 | | | | | 1 | | | | 2 | | | | 2 | | | | 2 | | | | 1 | | | | 2 | | | | 2 | | | | 2 | | | | 1 | | | | 2 | | | | 1 | | | | 2 | | | | 1 | | | | 2 | | | | 1 | |
| 168 | | | | 2 | | 1 | | 2 | | | | | | 2 | | | | 2 | | | | | | 2 | | | | 1 | | | | | | 1 | | | | 2 | | | | | | 1 | | | | 2 | | | | | 1 | | | 2 | | | | | 1 | | | 1 | | | | | 1 | | | 1 | | | | | | 1 | | | 1 | | | | | | 1 | | | 2 | | | | | | 2 | | | 1 | | | | | | 1 | | | 2 | | | | | | 1 | | | 1 | | | | | | 1 | | | 2 | | | | | | 1 | | | 2 | | | | | | 2 | | | 2 | | | | | | 1 | | | 2 | | | | | | 1 | | | 2 | | | | | | 1 | | | 1 | | | | | 1 | | | 2 | | | | | 2 | | | 2 | | | | | 1 | | | 1 | | | | | 1 | | | | 2 | | | | 2 | | | | 2 | | | | 1 | | | | 1 | | | | 1 | | | | 1 | | | | 1 | | | | 1 | | | | 1 | | | | 2 | | | | 2 | | | | 2 | | | | 1 | |
| 169 | | | | 2 | | 1 | | 2 | | | | | | 1 | | | | 2 | | | | | | 1 | | | | 2 | | | | | | 2 | | | | 2 | | | | | | 2 | | | | 2 | | | | | 1 | | | 2 | | | | | 2 | | | 2 | | | | | 1 | | | 2 | | | | | | 2 | | | 1 | | | | | | 1 | | | 2 | | | | | | 2 | | | 1 | | | | | | 1 | | | 2 | | | | | | 1 | | | 2 | | | | | | 1 | | | 2 | | | | | | 2 | | | 2 | | | | | | 1 | | | 2 | | | | | | 2 | | | 2 | | | | | | 2 | | | 1 | | | | | | 1 | | | 2 | | | | | 1 | | | 2 | | | | | 2 | | | 2 | | | | | 2 | | | 1 | | | | | 1 | | | | 2 | | | | 1 | | | | 2 | | | | 1 | | | | 2 | | | | 2 | | | | 2 | | | | 2 | | | | 2 | | | | 1 | | | | 2 | | | | 1 | | | | 2 | | | | 1 | |
| 170 | | | | 2 | | 1 | | 2 | | | | | | 2 | | | | 2 | | | | | | 1 | | | | 2 | | | | | | 2 | | | | 2 | | | | | | 2 | | | | 2 | | | | | 2 | | | 2 | | | | | 2 | | | 2 | | | | | 1 | | | 1 | | | | | | 1 | | | 1 | | | | | | 1 | | | 2 | | | | | | 2 | | | 1 | | | | | | 1 | | | 2 | | | | | | 2 | | | 1 | | | | | | 1 | | | 2 | | | | | | 1 | | | 1 | | | | | | 1 | | | 2 | | | | | | 2 | | | 2 | | | | | | 1 | | | 1 | | | | | | 1 | | | 2 | | | | | 2 | | | 2 | | | | | 1 | | | 2 | | | | | 2 | | | 1 | | | | | 1 | | | | 2 | | | | 2 | | | | 2 | | | | 2 | | | | 2 | | | | 2 | | | | 2 | | | | 2 | | | | 2 | | | | 1 | | | | 2 | | | | 1 | | | | 1 | | | | 1 | |
| 171 | | | | 1 | | 1 | | 2 | | | | | | 2 | | | | 1 | | | | | | 1 | | | | 2 | | | | | | 1 | | | | 2 | | | | | | 1 | | | | 2 | | | | | 2 | | | 1 | | | | | 1 | | | 1 | | | | | 1 | | | 1 | | | | | | 1 | | | 2 | | | | | | 1 | | | 2 | | | | | | 1 | | | 2 | | | | | | 1 | | | 1 | | | | | | 1 | | | 1 | | | | | | 1 | | | 2 | | | | | | 1 | | | 2 | | | | | | 1 | | | 2 | | | | | | 2 | | | 1 | | | | | | 1 | | | 1 | | | | | | 1 | | | 2 | | | | | 2 | | | 2 | | | | | 2 | | | 2 | | | | | 2 | | | 1 | | | | | 1 | | | | 2 | | | | 1 | | | | 2 | | | | 1 | | | | 2 | | | | 2 | | | | 2 | | | | 1 | | | | 1 | | | | 1 | | | | 2 | | | | 2 | | | | 2 | | | | 1 | |
| 172 | | | | 2 | | 1 | | 2 | | | | | | 1 | | | | 2 | | | | | | 2 | | | | 2 | | | | | | 1 | | | | 2 | | | | | | 1 | | | | 1 | | | | | 1 | | | 1 | | | | | 1 | | | 1 | | | | | 1 | | | 1 | | | | | | 1 | | | 1 | | | | | | 1 | | | 2 | | | | | | 1 | | | 1 | | | | | | 1 | | | 2 | | | | | | 1 | | | 2 | | | | | | 1 | | | 2 | | | | | | 1 | | | 2 | | | | | | 1 | | | 1 | | | | | | 1 | | | 2 | | | | | | 1 | | | 2 | | | | | | 1 | | | 2 | | | | | 2 | | | 2 | | | | | 2 | | | 2 | | | | | 2 | | | 1 | | | | | 1 | | | | 2 | | | | 2 | | | | 1 | | | | 1 | | | | 1 | | | | 1 | | | | 2 | | | | 1 | | | | 2 | | | | 2 | | | | 2 | | | | 2 | | | | 2 | | | | 1 | |
| 173 | | | | 2 | | 1 | | 2 | | | | | | 1 | | | | 1 | | | | | | 1 | | | | 2 | | | | | | 2 | | | | 2 | | | | | | 1 | | | | 2 | | | | | 2 | | | 2 | | | | | 1 | | | 1 | | | | | 1 | | | 1 | | | | | | 1 | | | 1 | | | | | | 1 | | | 2 | | | | | | 1 | | | 1 | | | | | | 1 | | | 2 | | | | | | 2 | | | 2 | | | | | | 1 | | | 2 | | | | | | 1 | | | 1 | | | | | | 1 | | | 2 | | | | | | 2 | | | 2 | | | | | | 2 | | | 2 | | | | | | 1 | | | 2 | | | | | 1 | | | 2 | | | | | 2 | | | 2 | | | | | 2 | | | 1 | | | | | 1 | | | | 2 | | | | 1 | | | | 2 | | | | 1 | | | | 1 | | | | 1 | | | | 2 | | | | 2 | | | | 2 | | | | 2 | | | | 2 | | | | 1 | | | | 2 | | | | 1 | |
| 174 | | | | 1 | | 1 | | 2 | | | | | | 2 | | | | 1 | | | | | | 1 | | | | 2 | | | | | | 1 | | | | 2 | | | | | | 2 | | | | 2 | | | | | 1 | | | 2 | | | | | 1 | | | 1 | | | | | 1 | | | 2 | | | | | | 1 | | | 1 | | | | | | 1 | | | 2 | | | | | | 1 | | | 2 | | | | | | 2 | | | 2 | | | | | | 2 | | | 2 | | | | | | 1 | | | 1 | | | | | | 1 | | | 2 | | | | | | 2 | | | 1 | | | | | | 1 | | | 2 | | | | | | 2 | | | 2 | | | | | | 2 | | | 2 | | | | | 1 | | | 2 | | | | | 2 | | | 2 | | | | | 2 | | | 2 | | | | | 1 | | | | 2 | | | | 1 | | | | 1 | | | | 1 | | | | 2 | | | | 1 | | | | 2 | | | | 1 | | | | 1 | | | | 1 | | | | 2 | | | | 2 | | | | 2 | | | | 1 | |
| 175 | | | | 2 | | 1 | | 2 | | | | | | 2 | | | | 2 | | | | | | 2 | | | | 1 | | | | | | 1 | | | | 2 | | | | | | 1 | | | | 2 | | | | | 2 | | | 2 | | | | | 2 | | | 2 | | | | | 1 | | | 2 | | | | | | 2 | | | 2 | | | | | | 1 | | | 2 | | | | | | 1 | | | 2 | | | | | | 1 | | | 1 | | | | | | 1 | | | 2 | | | | | | 1 | | | 2 | | | | | | 2 | | | 2 | | | | | | 1 | | | 2 | | | | | | 1 | | | 2 | | | | | | 1 | | | 2 | | | | | | 2 | | | 2 | | | | | 2 | | | 2 | | | | | 2 | | | 2 | | | | | 2 | | | 1 | | | | | 1 | | | | 2 | | | | 2 | | | | 2 | | | | 1 | | | | 2 | | | | 1 | | | | 2 | | | | 2 | | | | 1 | | | | 1 | | | | 2 | | | | 2 | | | | 2 | | | | 1 | |
| 176 | | | | 1 | | 1 | | 2 | | | | | | 1 | | | | 2 | | | | | | 1 | | | | 1 | | | | | | 1 | | | | 2 | | | | | | 2 | | | | 1 | | | | | 1 | | | 2 | | | | | 1 | | | 2 | | | | | 1 | | | 2 | | | | | | 1 | | | 2 | | | | | | 1 | | | 2 | | | | | | 1 | | | 1 | | | | | | 1 | | | 1 | | | | | | 1 | | | 1 | | | | | | 1 | | | 2 | | | | | | 1 | | | 2 | | | | | | 1 | | | 1 | | | | | | 1 | | | 2 | | | | | | 2 | | | 1 | | | | | | 1 | | | 2 | | | | | 1 | | | 2 | | | | | 2 | | | 2 | | | | | 2 | | | 1 | | | | | 1 | | | | 2 | | | | 1 | | | | 2 | | | | 1 | | | | 2 | | | | 1 | | | | 2 | | | | 2 | | | | 2 | | | | 1 | | | | 2 | | | | 2 | | | | 1 | | | | 1 | |
| 177 | | | | 1 | | 1 | | 2 | | | | | | 2 | | | | 2 | | | | | | 1 | | | | 1 | | | | | | 1 | | | | 2 | | | | | | 1 | | | | 1 | | | | | 1 | | | 2 | | | | | 1 | | | 2 | | | | | 1 | | | 1 | | | | | | 1 | | | 2 | | | | | | 1 | | | 2 | | | | | | 2 | | | 1 | | | | | | 1 | | | 2 | | | | | | 2 | | | 2 | | | | | | 1 | | | 2 | | | | | | 2 | | | 2 | | | | | | 1 | | | 2 | | | | | | 1 | | | 2 | | | | | | 2 | | | 1 | | | | | | 1 | | | 1 | | | | | 1 | | | 2 | | | | | 2 | | | 2 | | | | | 2 | | | 1 | | | | | 1 | | | | 2 | | | | 2 | | | | 2 | | | | 1 | | | | 2 | | | | 1 | | | | 2 | | | | 1 | | | | 2 | | | | 1 | | | | 2 | | | | 2 | | | | 2 | | | | 1 | |
| 178 | | | | 1 | | 1 | | 2 | | | | | | 1 | | | | 1 | | | | | | 1 | | | | 1 | | | | | | 1 | | | | 2 | | | | | | 2 | | | | 2 | | | | | 1 | | | 2 | | | | | 2 | | | 1 | | | | | 1 | | | 1 | | | | | | 1 | | | 1 | | | | | | 1 | | | 2 | | | | | | 1 | | | 2 | | | | | | 1 | | | 2 | | | | | | 2 | | | 2 | | | | | | 1 | | | 2 | | | | | | 1 | | | 2 | | | | | | 2 | | | 2 | | | | | | 1 | | | 2 | | | | | | 1 | | | 1 | | | | | | 1 | | | 2 | | | | | 2 | | | 2 | | | | | 2 | | | 2 | | | | | 1 | | | 2 | | | | | 1 | | | | 2 | | | | 2 | | | | 2 | | | | 2 | | | | 1 | | | | 1 | | | | 2 | | | | 1 | | | | 1 | | | | 1 | | | | 2 | | | | 1 | | | | 2 | | | | 1 | |
| 180 | | | | 2 | | 2 | | 2 | | | | | | 2 | | | | 1 | | | | | | 1 | | | | 1 | | | | | | 1 | | | | 2 | | | | | | 2 | | | | 1 | | | | | 1 | | | 1 | | | | | 1 | | | 1 | | | | | 1 | | | 2 | | | | | | 1 | | | 2 | | | | | | 1 | | | 2 | | | | | | 2 | | | 1 | | | | | | 1 | | | 2 | | | | | | 1 | | | 2 | | | | | | 1 | | | 2 | | | | | | 1 | | | 2 | | | | | | 2 | | | 2 | | | | | | 1 | | | 1 | | | | | | 1 | | | 2 | | | | | | 1 | | | 2 | | | | | 1 | | | 2 | | | | | 1 | | | 2 | | | | | 2 | | | 1 | | | | | 1 | | | | 2 | | | | 1 | | | | 2 | | | | 1 | | | | 2 | | | | 1 | | | | 2 | | | | 1 | | | | 2 | | | | 1 | | | | 2 | | | | 2 | | | | 1 | | | | 1 | |
| 181 | | | | 2 | | 2 | | 2 | | | | | | 2 | | | | 2 | | | | | | 1 | | | | 1 | | | | | | 1 | | | | 2 | | | | | | 2 | | | | 2 | | | | | 1 | | | 2 | | | | | 1 | | | 1 | | | | | 1 | | | 1 | | | | | | 1 | | | 1 | | | | | | 1 | | | 2 | | | | | | 2 | | | 1 | | | | | | 1 | | | 2 | | | | | | 1 | | | 2 | | | | | | 1 | | | 1 | | | | | | 1 | | | 2 | | | | | | 2 | | | 1 | | | | | | 1 | | | 2 | | | | | | 1 | | | 1 | | | | | | 1 | | | 2 | | | | | 1 | | | 2 | | | | | 2 | | | 2 | | | | | 1 | | | 1 | | | | | 1 | | | | 2 | | | | 2 | | | | 1 | | | | 1 | | | | 2 | | | | 2 | | | | 2 | | | | 2 | | | | 2 | | | | 1 | | | | 2 | | | | 2 | | | | 2 | | | | 2 | |
| 182 | | | | 2 | | 1 | | 2 | | | | | | 2 | | | | 2 | | | | | | 1 | | | | 2 | | | | | | 1 | | | | 2 | | | | | | 1 | | | | 2 | | | | | 1 | | | 2 | | | | | 2 | | | 2 | | | | | 1 | | | 1 | | | | | | 1 | | | 2 | | | | | | 1 | | | 2 | | | | | | 2 | | | 1 | | | | | | 1 | | | 1 | | | | | | 1 | | | 1 | | | | | | 1 | | | 1 | | | | | | 1 | | | 1 | | | | | | 1 | | | 1 | | | | | | 1 | | | 2 | | | | | | 2 | | | 1 | | | | | | 1 | | | 2 | | | | | 1 | | | 2 | | | | | 2 | | | 2 | | | | | 1 | | | 2 | | | | | 1 | | | | 2 | | | | 2 | | | | 2 | | | | 2 | | | | 1 | | | | 1 | | | | 2 | | | | 2 | | | | 2 | | | | 1 | | | | 2 | | | | 1 | | | | 2 | | | | 1 | |
| 183 | | | | 1 | | 1 | | 2 | | | | | | 1 | | | | 1 | | | | | | 1 | | | | 2 | | | | | | 1 | | | | 2 | | | | | | 2 | | | | 1 | | | | | 1 | | | 2 | | | | | 1 | | | 1 | | | | | 1 | | | 2 | | | | | | 1 | | | 1 | | | | | | 1 | | | 2 | | | | | | 2 | | | 2 | | | | | | 1 | | | 2 | | | | | | 1 | | | 2 | | | | | | 1 | | | 2 | | | | | | 2 | | | 1 | | | | | | 1 | | | 1 | | | | | | 1 | | | 2 | | | | | | 2 | | | 1 | | | | | | 1 | | | 1 | | | | | 1 | | | 2 | | | | | 2 | | | 2 | | | | | 1 | | | 1 | | | | | 1 | | | | 1 | | | | 1 | | | | 1 | | | | 1 | | | | 1 | | | | 1 | | | | 2 | | | | 1 | | | | 2 | | | | 2 | | | | 2 | | | | 1 | | | | 2 | | | | 2 | |
| 185 | | | | 2 | | 1 | | 2 | | | | | | 2 | | | | 1 | | | | | | 1 | | | | 2 | | | | | | 1 | | | | 2 | | | | | | 1 | | | | 1 | | | | | 1 | | | 2 | | | | | 1 | | | 2 | | | | | 2 | | | 2 | | | | | | 1 | | | 2 | | | | | | 1 | | | 2 | | | | | | 2 | | | 2 | | | | | | 2 | | | 2 | | | | | | 2 | | | 2 | | | | | | 1 | | | 1 | | | | | | 1 | | | 2 | | | | | | 1 | | | 2 | | | | | | 1 | | | 2 | | | | | | 1 | | | 2 | | | | | | 1 | | | 1 | | | | | 1 | | | 2 | | | | | 2 | | | 2 | | | | | 2 | | | 1 | | | | | 1 | | | | 2 | | | | 1 | | | | 1 | | | | 1 | | | | 2 | | | | 1 | | | | 2 | | | | 2 | | | | 2 | | | | 2 | | | | 2 | | | | 1 | | | | 2 | | | | 1 | |
| 186 | | | | 2 | | 1 | | 2 | | | | | | 1 | | | | 1 | | | | | | 1 | | | | 2 | | | | | | 1 | | | | 2 | | | | | | 1 | | | | 1 | | | | | 1 | | | 1 | | | | | 1 | | | 1 | | | | | 1 | | | 1 | | | | | | 1 | | | 2 | | | | | | 2 | | | 2 | | | | | | 2 | | | 1 | | | | | | 1 | | | 1 | | | | | | 1 | | | 2 | | | | | | 1 | | | 2 | | | | | | 1 | | | 2 | | | | | | 2 | | | 2 | | | | | | 2 | | | 2 | | | | | | 2 | | | 2 | | | | | | 1 | | | 2 | | | | | 2 | | | 2 | | | | | 1 | | | 2 | | | | | 2 | | | 2 | | | | | 1 | | | | 2 | | | | 2 | | | | 2 | | | | 1 | | | | 2 | | | | 1 | | | | 2 | | | | 1 | | | | 2 | | | | 1 | | | | 2 | | | | 2 | | | | 2 | | | | 1 | |
| 187 | | | | 2 | | 1 | | 2 | | | | | | 2 | | | | 1 | | | | | | 1 | | | | 2 | | | | | | 1 | | | | 2 | | | | | | 2 | | | | 2 | | | | | 2 | | | 2 | | | | | 2 | | | 1 | | | | | 1 | | | 2 | | | | | | 1 | | | 2 | | | | | | 2 | | | 1 | | | | | | 1 | | | 2 | | | | | | 1 | | | 2 | | | | | | 1 | | | 2 | | | | | | 1 | | | 1 | | | | | | 1 | | | 2 | | | | | | 1 | | | 2 | | | | | | 2 | | | 2 | | | | | | 1 | | | 2 | | | | | | 1 | | | 2 | | | | | 1 | | | 2 | | | | | 2 | | | 2 | | | | | 1 | | | 1 | | | | | 1 | | | | 2 | | | | 2 | | | | 1 | | | | 1 | | | | 2 | | | | 1 | | | | 2 | | | | 1 | | | | 2 | | | | 1 | | | | 2 | | | | 2 | | | | 2 | | | | 1 | |
| 189 | | | | 1 | | 1 | | 2 | | | | | | 2 | | | | 2 | | | | | | 1 | | | | 2 | | | | | | 1 | | | | 2 | | | | | | 1 | | | | 2 | | | | | 1 | | | 1 | | | | | 1 | | | 2 | | | | | 1 | | | 1 | | | | | | 1 | | | 1 | | | | | | 1 | | | 2 | | | | | | 1 | | | 1 | | | | | | 1 | | | 2 | | | | | | 1 | | | 2 | | | | | | 2 | | | 1 | | | | | | 1 | | | 2 | | | | | | 1 | | | 2 | | | | | | 1 | | | 2 | | | | | | 1 | | | 2 | | | | | | 1 | | | 2 | | | | | 2 | | | 2 | | | | | 2 | | | 2 | | | | | 2 | | | 1 | | | | | 1 | | | | 2 | | | | 2 | | | | 2 | | | | 1 | | | | 2 | | | | 1 | | | | 1 | | | | 1 | | | | 2 | | | | 1 | | | | 2 | | | | 1 | | | | 2 | | | | 1 | |
| 190 | | | | 1 | | 1 | | 2 | | | | | | 2 | | | | 2 | | | | | | 1 | | | | 2 | | | | | | 1 | | | | 2 | | | | | | 2 | | | | 2 | | | | | 1 | | | 2 | | | | | 1 | | | 1 | | | | | 1 | | | 1 | | | | | | 1 | | | 1 | | | | | | 1 | | | 2 | | | | | | 1 | | | 1 | | | | | | 1 | | | 2 | | | | | | 2 | | | 2 | | | | | | 1 | | | 1 | | | | | | 1 | | | 2 | | | | | | 2 | | | 2 | | | | | | 1 | | | 2 | | | | | | 2 | | | 1 | | | | | | 1 | | | 2 | | | | | 2 | | | 2 | | | | | 2 | | | 2 | | | | | 1 | | | 1 | | | | | 1 | | | | 2 | | | | 2 | | | | 1 | | | | 1 | | | | 1 | | | | 1 | | | | 1 | | | | 1 | | | | 2 | | | | 1 | | | | 2 | | | | 2 | | | | 2 | | | | 1 | |
| 191 | | | | 2 | | 1 | | 2 | | | | | | 2 | | | | 2 | | | | | | 1 | | | | 1 | | | | | | 1 | | | | 2 | | | | | | 2 | | | | 2 | | | | | 1 | | | 2 | | | | | 2 | | | 1 | | | | | 1 | | | 1 | | | | | | 1 | | | 1 | | | | | | 1 | | | 2 | | | | | | 1 | | | 1 | | | | | | 1 | | | 2 | | | | | | 1 | | | 2 | | | | | | 1 | | | 2 | | | | | | 2 | | | 2 | | | | | | 1 | | | 1 | | | | | | 1 | | | 2 | | | | | | 2 | | | 1 | | | | | | 1 | | | 2 | | | | | 1 | | | 2 | | | | | 2 | | | 2 | | | | | 1 | | | 1 | | | | | 1 | | | | 2 | | | | 1 | | | | 2 | | | | 1 | | | | 1 | | | | 1 | | | | 2 | | | | 2 | | | | 2 | | | | 1 | | | | 2 | | | | 2 | | | | 2 | | | | 2 | |
| Sample ID  *Supplementary Table (Continued)* | HLD  6 | | | | | | HLD  39 | | | | | | | | | | | | | | HLD  40 | | | | | | | | | | HLD  45 | | | | | | | | | | HLD  48 | | | | | | | | HLD  56 | | | | | | | | HLD  58 | | | | | | | | HLD  64 | | | | | | | | HLD  67 | | | | | | | | | HLD  70 | | | | | | | | | HLD  77 | | | | | | | | | HLD  81 | | | | | | | | | HLD  83 | | | | | | | | | HLD  84 | | | | | | | | | HLD  88 | | | | | | | | | HLD  92 | | | | | | | | | HLD  93 | | | | | | | | | HLD  97 | | | | | | | | | HLD  99 | | | | | | | | | HLD  101 | | | | | | | | HLD  111 | | | | | | | | HLD  114 | | | | | | | | HLD  118 | | | | | | HLD  122 | | | | | | | | HLD  124 | | | | | | | HLD  125 | | | | | | | | HLD  128 | | | | | | | | HLD  131 | | | | | | | | HLD  133 | | | | | | | | HLD  136 | | | | | | | |  |
| 192 | | | 2 | | 1 | | 2 | | | | | | 1 | | | | 1 | | | | | | 1 | | | | 1 | | | | | | 1 | | | | 2 | | | | | | 1 | | | 2 | | | | | 1 | | | 2 | | | | | 1 | | | 1 | | | | | 1 | | | 1 | | | | | | 1 | | | 2 | | | | | | 1 | | | 2 | | | | | | 1 | | | 1 | | | | | | 1 | | | 2 | | | | | | 1 | | | 1 | | | | | | 1 | | | 2 | | | | | | 1 | | | 2 | | | | | | 2 | | | 2 | | | | | | 2 | | | 2 | | | | | | 2 | | | 2 | | | | | | 1 | | | 2 | | | | | 1 | | | 2 | | | | | 2 | | | 2 | | | | | 1 | | | 1 | | | | | 1 | | | 2 | | | | | 2 | | | 2 | | | | | 2 | | | | 2 | | | | 2 | | | | 2 | | | | 1 | | | | 1 | | | | 1 | | | | 2 | | | | 1 | | | | 2 | | | | 1 | | | |
| 193 | | | 2 | | 2 | | 2 | | | | | | 2 | | | | 1 | | | | | | 1 | | | | 1 | | | | | | 1 | | | | 2 | | | | | | 1 | | | 2 | | | | | 2 | | | 2 | | | | | 1 | | | 2 | | | | | 1 | | | 2 | | | | | | 1 | | | 2 | | | | | | 1 | | | 2 | | | | | | 1 | | | 1 | | | | | | 1 | | | 2 | | | | | | 1 | | | 2 | | | | | | 1 | | | 2 | | | | | | 1 | | | 2 | | | | | | 1 | | | 2 | | | | | | 1 | | | 2 | | | | | | 1 | | | 1 | | | | | | 1 | | | 2 | | | | | 2 | | | 2 | | | | | 2 | | | 2 | | | | | 2 | | | 1 | | | | | 1 | | | 2 | | | | | 2 | | | 2 | | | | | 1 | | | | 1 | | | | 1 | | | | 2 | | | | 1 | | | | 2 | | | | 2 | | | | 2 | | | | 2 | | | | 1 | | | | 1 | | | |
| 194 | | | 2 | | 1 | | 2 | | | | | | 1 | | | | 2 | | | | | | 1 | | | | 1 | | | | | | 1 | | | | 1 | | | | | | 1 | | | 2 | | | | | 1 | | | 2 | | | | | 2 | | | 1 | | | | | 1 | | | 1 | | | | | | 1 | | | 1 | | | | | | 1 | | | 1 | | | | | | 1 | | | 1 | | | | | | 1 | | | 2 | | | | | | 1 | | | 2 | | | | | | 1 | | | 2 | | | | | | 1 | | | 1 | | | | | | 1 | | | 2 | | | | | | 1 | | | 2 | | | | | | 1 | | | 1 | | | | | | 1 | | | 2 | | | | | 1 | | | 2 | | | | | 2 | | | 2 | | | | | 1 | | | 1 | | | | | 1 | | | 2 | | | | | 2 | | | 2 | | | | | 1 | | | | 2 | | | | 2 | | | | 1 | | | | 1 | | | | 2 | | | | 2 | | | | 2 | | | | 1 | | | | 2 | | | | 1 | | | |
| 195 | | | 2 | | 2 | | 2 | | | | | | 2 | | | | 2 | | | | | | 1 | | | | 1 | | | | | | 1 | | | | 2 | | | | | | 1 | | | 1 | | | | | 1 | | | 1 | | | | | 1 | | | 1 | | | | | 1 | | | 1 | | | | | | 1 | | | 1 | | | | | | 1 | | | 1 | | | | | | 1 | | | 1 | | | | | | 1 | | | 2 | | | | | | 2 | | | 2 | | | | | | 2 | | | 2 | | | | | | 1 | | | 1 | | | | | | 1 | | | 2 | | | | | | 2 | | | 2 | | | | | | 2 | | | 1 | | | | | | 1 | | | 2 | | | | | 2 | | | 2 | | | | | 2 | | | 2 | | | | | 2 | | | 1 | | | | | 1 | | | 2 | | | | | 1 | | | 2 | | | | | 1 | | | | 2 | | | | 1 | | | | 2 | | | | 2 | | | | 2 | | | | 1 | | | | 1 | | | | 1 | | | | 1 | | | | 1 | | | |
| 197 | | | 2 | | 1 | | 2 | | | | | | 1 | | | | 1 | | | | | | 1 | | | | 2 | | | | | | 1 | | | | 2 | | | | | | 2 | | | 2 | | | | | 1 | | | 2 | | | | | 1 | | | 2 | | | | | 1 | | | 2 | | | | | | 1 | | | 2 | | | | | | 1 | | | 2 | | | | | | 1 | | | 1 | | | | | | 1 | | | 2 | | | | | | 1 | | | 1 | | | | | | 1 | | | 2 | | | | | | 2 | | | 1 | | | | | | 1 | | | 2 | | | | | | 1 | | | 1 | | | | | | 1 | | | 1 | | | | | | 1 | | | 2 | | | | | 2 | | | 2 | | | | | 2 | | | 2 | | | | | 2 | | | 2 | | | | | 1 | | | 1 | | | | | 1 | | | 2 | | | | | 1 | | | | 2 | | | | 1 | | | | 2 | | | | 2 | | | | 2 | | | | 2 | | | | 2 | | | | 1 | | | | 2 | | | | 1 | | | |
| 198 | | | 2 | | 1 | | 2 | | | | | | 2 | | | | 1 | | | | | | 1 | | | | 2 | | | | | | 1 | | | | 2 | | | | | | 2 | | | 2 | | | | | 2 | | | 2 | | | | | 1 | | | 1 | | | | | 1 | | | 2 | | | | | | 1 | | | 2 | | | | | | 2 | | | 2 | | | | | | 2 | | | 1 | | | | | | 1 | | | 2 | | | | | | 1 | | | 1 | | | | | | 1 | | | 2 | | | | | | 1 | | | 2 | | | | | | 1 | | | 1 | | | | | | 1 | | | 2 | | | | | | 1 | | | 2 | | | | | | 1 | | | 2 | | | | | 1 | | | 2 | | | | | 2 | | | 2 | | | | | 1 | | | 1 | | | | | 1 | | | 2 | | | | | 2 | | | 2 | | | | | 2 | | | | 2 | | | | 1 | | | | 2 | | | | 2 | | | | 2 | | | | 1 | | | | 2 | | | | 1 | | | | 1 | | | | 1 | | | |
| 199 | | | 2 | | 2 | | 2 | | | | | | 2 | | | | 1 | | | | | | 1 | | | | 1 | | | | | | 1 | | | | 2 | | | | | | 1 | | | 2 | | | | | 1 | | | 2 | | | | | 1 | | | 1 | | | | | 1 | | | 2 | | | | | | 2 | | | 1 | | | | | | 1 | | | 1 | | | | | | 1 | | | 1 | | | | | | 1 | | | 2 | | | | | | 1 | | | 1 | | | | | | 1 | | | 2 | | | | | | 1 | | | 2 | | | | | | 2 | | | 1 | | | | | | 1 | | | 2 | | | | | | 2 | | | 2 | | | | | | 1 | | | 1 | | | | | 1 | | | 2 | | | | | 1 | | | 2 | | | | | 2 | | | 1 | | | | | 1 | | | 2 | | | | | 2 | | | 2 | | | | | 2 | | | | 2 | | | | 1 | | | | 2 | | | | 2 | | | | 2 | | | | 2 | | | | 2 | | | | 1 | | | | 2 | | | | 1 | | | |
| 200 | | | 1 | | 1 | | 2 | | | | | | 2 | | | | 2 | | | | | | 2 | | | | 2 | | | | | | 1 | | | | 2 | | | | | | 1 | | | 2 | | | | | 1 | | | 2 | | | | | 1 | | | 2 | | | | | 1 | | | 1 | | | | | | 1 | | | 1 | | | | | | 1 | | | 2 | | | | | | 2 | | | 1 | | | | | | 1 | | | 2 | | | | | | 1 | | | 1 | | | | | | 1 | | | 1 | | | | | | 1 | | | 2 | | | | | | 1 | | | 2 | | | | | | 1 | | | 2 | | | | | | 2 | | | 1 | | | | | | 1 | | | 1 | | | | | 1 | | | 2 | | | | | 1 | | | 2 | | | | | 2 | | | 1 | | | | | 1 | | | 2 | | | | | 2 | | | 1 | | | | | 1 | | | | 2 | | | | 2 | | | | 2 | | | | 1 | | | | 1 | | | | 1 | | | | 1 | | | | 1 | | | | 2 | | | | 2 | | | |
| 201 | | | 2 | | 2 | | 2 | | | | | | 2 | | | | 2 | | | | | | 2 | | | | 2 | | | | | | 1 | | | | 2 | | | | | | 1 | | | 1 | | | | | 1 | | | 1 | | | | | 1 | | | 1 | | | | | 1 | | | 2 | | | | | | 2 | | | 2 | | | | | | 1 | | | 2 | | | | | | 1 | | | 2 | | | | | | 2 | | | 2 | | | | | | 1 | | | 1 | | | | | | 1 | | | 2 | | | | | | 1 | | | 1 | | | | | | 1 | | | 1 | | | | | | 1 | | | 2 | | | | | | 2 | | | 1 | | | | | | 1 | | | 1 | | | | | 1 | | | 2 | | | | | 1 | | | 2 | | | | | 2 | | | 1 | | | | | 1 | | | 2 | | | | | 1 | | | 2 | | | | | 1 | | | | 2 | | | | 1 | | | | 2 | | | | 1 | | | | 2 | | | | 1 | | | | 2 | | | | 1 | | | | 1 | | | | 1 | | | |
| 202 | | | 2 | | 1 | | 2 | | | | | | 2 | | | | 1 | | | | | | 1 | | | | 2 | | | | | | 1 | | | | 2 | | | | | | 1 | | | 1 | | | | | 1 | | | 2 | | | | | 1 | | | 1 | | | | | 1 | | | 2 | | | | | | 1 | | | 2 | | | | | | 1 | | | 2 | | | | | | 1 | | | 1 | | | | | | 1 | | | 2 | | | | | | 1 | | | 1 | | | | | | 1 | | | 1 | | | | | | 1 | | | 2 | | | | | | 2 | | | 2 | | | | | | 2 | | | 2 | | | | | | 2 | | | 1 | | | | | | 1 | | | 1 | | | | | 1 | | | 2 | | | | | 1 | | | 2 | | | | | 1 | | | 1 | | | | | 1 | | | 2 | | | | | 1 | | | 2 | | | | | 1 | | | | 2 | | | | 2 | | | | 2 | | | | 2 | | | | 2 | | | | 1 | | | | 2 | | | | 1 | | | | 1 | | | | 1 | | | |
| 203 | | | 1 | | 1 | | 2 | | | | | | 2 | | | | 1 | | | | | | 1 | | | | 1 | | | | | | 1 | | | | 2 | | | | | | 1 | | | 2 | | | | | 1 | | | 2 | | | | | 1 | | | 1 | | | | | 1 | | | 2 | | | | | | 1 | | | 2 | | | | | | 1 | | | 2 | | | | | | 1 | | | 2 | | | | | | 1 | | | 2 | | | | | | 1 | | | 2 | | | | | | 1 | | | 2 | | | | | | 1 | | | 1 | | | | | | 1 | | | 1 | | | | | | 1 | | | 2 | | | | | | 2 | | | 1 | | | | | | 1 | | | 1 | | | | | 1 | | | 2 | | | | | 2 | | | 2 | | | | | 2 | | | 1 | | | | | 1 | | | 2 | | | | | 2 | | | 2 | | | | | 1 | | | | 2 | | | | 2 | | | | 2 | | | | 2 | | | | 2 | | | | 1 | | | | 2 | | | | 1 | | | | 2 | | | | 1 | | | |
| 204 | | | 2 | | 1 | | 2 | | | | | | 2 | | | | 1 | | | | | | 1 | | | | 1 | | | | | | 1 | | | | 2 | | | | | | 1 | | | 2 | | | | | 1 | | | 2 | | | | | 1 | | | 1 | | | | | 1 | | | 2 | | | | | | 1 | | | 2 | | | | | | 1 | | | 1 | | | | | | 1 | | | 2 | | | | | | 1 | | | 2 | | | | | | 1 | | | 1 | | | | | | 1 | | | 2 | | | | | | 1 | | | 2 | | | | | | 1 | | | 2 | | | | | | 2 | | | 1 | | | | | | 1 | | | 1 | | | | | | 1 | | | 2 | | | | | 2 | | | 2 | | | | | 2 | | | 2 | | | | | 2 | | | 1 | | | | | 1 | | | 2 | | | | | 1 | | | 1 | | | | | 1 | | | | 2 | | | | 2 | | | | 2 | | | | 2 | | | | 2 | | | | 1 | | | | 2 | | | | 2 | | | | 2 | | | | 1 | | | |
| 205 | | | 1 | | 1 | | 2 | | | | | | 2 | | | | 1 | | | | | | 1 | | | | 2 | | | | | | 2 | | | | 2 | | | | | | 1 | | | 2 | | | | | 1 | | | 2 | | | | | 2 | | | 1 | | | | | 1 | | | 1 | | | | | | 1 | | | 1 | | | | | | 1 | | | 1 | | | | | | 1 | | | 1 | | | | | | 1 | | | 1 | | | | | | 1 | | | 1 | | | | | | 1 | | | 2 | | | | | | 1 | | | 2 | | | | | | 1 | | | 1 | | | | | | 1 | | | 1 | | | | | | 1 | | | 2 | | | | | | 1 | | | 2 | | | | | 1 | | | 2 | | | | | 1 | | | 2 | | | | | 2 | | | 2 | | | | | 2 | | | 2 | | | | | 1 | | | 1 | | | | | 1 | | | | 1 | | | | 1 | | | | 2 | | | | 2 | | | | 2 | | | | 1 | | | | 2 | | | | 1 | | | | 1 | | | | 1 | | | |
| 206 | | | 2 | | 1 | | 2 | | | | | | 2 | | | | 1 | | | | | | 1 | | | | 2 | | | | | | 1 | | | | 2 | | | | | | 1 | | | 2 | | | | | 1 | | | 2 | | | | | 1 | | | 1 | | | | | 1 | | | 2 | | | | | | 1 | | | 1 | | | | | | 1 | | | 2 | | | | | | 2 | | | 1 | | | | | | 1 | | | 2 | | | | | | 1 | | | 2 | | | | | | 1 | | | 1 | | | | | | 1 | | | 2 | | | | | | 2 | | | 2 | | | | | | 1 | | | 2 | | | | | | 1 | | | 1 | | | | | | 1 | | | 2 | | | | | 2 | | | 2 | | | | | 2 | | | 2 | | | | | 2 | | | 1 | | | | | 1 | | | 2 | | | | | 2 | | | 2 | | | | | 2 | | | | 2 | | | | 1 | | | | 2 | | | | 2 | | | | 2 | | | | 1 | | | | 2 | | | | 1 | | | | 1 | | | | 1 | | | |
| 207 | | | 2 | | 2 | | 2 | | | | | | 1 | | | | 2 | | | | | | 1 | | | | 2 | | | | | | 1 | | | | 2 | | | | | | 2 | | | 2 | | | | | 2 | | | 2 | | | | | 1 | | | 1 | | | | | 1 | | | 1 | | | | | | 1 | | | 2 | | | | | | 1 | | | 2 | | | | | | 1 | | | 1 | | | | | | 1 | | | 2 | | | | | | 1 | | | 2 | | | | | | 1 | | | 1 | | | | | | 1 | | | 1 | | | | | | 1 | | | 1 | | | | | | 1 | | | 2 | | | | | | 2 | | | 1 | | | | | | 1 | | | 2 | | | | | 2 | | | 2 | | | | | 2 | | | 2 | | | | | 2 | | | 1 | | | | | 1 | | | 2 | | | | | 2 | | | 2 | | | | | 1 | | | | 2 | | | | 1 | | | | 2 | | | | 2 | | | | 2 | | | | 1 | | | | 2 | | | | 2 | | | | 2 | | | | 1 | | | |
| 208 | | | 2 | | 2 | | 2 | | | | | | 1 | | | | 1 | | | | | | 1 | | | | 2 | | | | | | 1 | | | | 2 | | | | | | 1 | | | 2 | | | | | 2 | | | 2 | | | | | 2 | | | 1 | | | | | 1 | | | 2 | | | | | | 1 | | | 1 | | | | | | 1 | | | 2 | | | | | | 1 | | | 1 | | | | | | 1 | | | 2 | | | | | | 2 | | | 2 | | | | | | 2 | | | 1 | | | | | | 1 | | | 2 | | | | | | 2 | | | 1 | | | | | | 1 | | | 2 | | | | | | 2 | | | 1 | | | | | | 1 | | | 1 | | | | | 1 | | | 2 | | | | | 2 | | | 2 | | | | | 2 | | | 1 | | | | | 1 | | | 2 | | | | | 1 | | | 2 | | | | | 1 | | | | 2 | | | | 2 | | | | 2 | | | | 1 | | | | 2 | | | | 1 | | | | 2 | | | | 1 | | | | 2 | | | | 2 | | | |
| 209 | | | 2 | | 1 | | 2 | | | | | | 1 | | | | 2 | | | | | | 1 | | | | 2 | | | | | | 1 | | | | 2 | | | | | | 1 | | | 1 | | | | | 1 | | | 1 | | | | | 1 | | | 2 | | | | | 1 | | | 1 | | | | | | 1 | | | 2 | | | | | | 1 | | | 2 | | | | | | 1 | | | 1 | | | | | | 1 | | | 2 | | | | | | 1 | | | 2 | | | | | | 1 | | | 1 | | | | | | 1 | | | 2 | | | | | | 1 | | | 1 | | | | | | 1 | | | 2 | | | | | | 2 | | | 1 | | | | | | 1 | | | 2 | | | | | 2 | | | 2 | | | | | 1 | | | 2 | | | | | 2 | | | 2 | | | | | 1 | | | 2 | | | | | 2 | | | 1 | | | | | 1 | | | | 2 | | | | 1 | | | | 1 | | | | 1 | | | | 2 | | | | 1 | | | | 2 | | | | 2 | | | | 2 | | | | 1 | | | |
| 210 | | | 2 | | 1 | | 2 | | | | | | 1 | | | | 2 | | | | | | 1 | | | | 1 | | | | | | 1 | | | | 2 | | | | | | 1 | | | 2 | | | | | 1 | | | 2 | | | | | 2 | | | 1 | | | | | 1 | | | 2 | | | | | | 1 | | | 2 | | | | | | 2 | | | 2 | | | | | | 1 | | | 1 | | | | | | 1 | | | 2 | | | | | | 1 | | | 1 | | | | | | 1 | | | 2 | | | | | | 2 | | | 2 | | | | | | 1 | | | 2 | | | | | | 1 | | | 2 | | | | | | 1 | | | 1 | | | | | | 1 | | | 1 | | | | | 1 | | | 2 | | | | | 2 | | | 2 | | | | | 2 | | | 1 | | | | | 1 | | | 2 | | | | | 1 | | | 2 | | | | | 1 | | | | 2 | | | | 1 | | | | 2 | | | | 2 | | | | 2 | | | | 1 | | | | 2 | | | | 1 | | | | 2 | | | | 1 | | | |
| 211 | | | 1 | | 1 | | 2 | | | | | | 2 | | | | 1 | | | | | | 1 | | | | 2 | | | | | | 1 | | | | 2 | | | | | | 2 | | | 2 | | | | | 1 | | | 2 | | | | | 2 | | | 1 | | | | | 1 | | | 2 | | | | | | 1 | | | 1 | | | | | | 1 | | | 1 | | | | | | 1 | | | 1 | | | | | | 1 | | | 2 | | | | | | 2 | | | 1 | | | | | | 1 | | | 2 | | | | | | 1 | | | 2 | | | | | | 1 | | | 2 | | | | | | 2 | | | 2 | | | | | | 2 | | | 1 | | | | | | 1 | | | 2 | | | | | 1 | | | 2 | | | | | 2 | | | 2 | | | | | 2 | | | 1 | | | | | 1 | | | 2 | | | | | 2 | | | 2 | | | | | 1 | | | | 2 | | | | 2 | | | | 2 | | | | 2 | | | | 2 | | | | 2 | | | | 2 | | | | 2 | | | | 1 | | | | 1 | | | |
| 212 | | | 2 | | 1 | | 2 | | | | | | 2 | | | | 2 | | | | | | 1 | | | | 2 | | | | | | 2 | | | | 2 | | | | | | 1 | | | 1 | | | | | 1 | | | 2 | | | | | 2 | | | 2 | | | | | 1 | | | 2 | | | | | | 1 | | | 2 | | | | | | 1 | | | 2 | | | | | | 1 | | | 1 | | | | | | 1 | | | 2 | | | | | | 2 | | | 2 | | | | | | 1 | | | 2 | | | | | | 1 | | | 2 | | | | | | 1 | | | 2 | | | | | | 1 | | | 2 | | | | | | 2 | | | 1 | | | | | | 1 | | | 1 | | | | | 1 | | | 2 | | | | | 1 | | | 2 | | | | | 2 | | | 1 | | | | | 1 | | | 2 | | | | | 1 | | | 2 | | | | | 2 | | | | 1 | | | | 1 | | | | 2 | | | | 1 | | | | 2 | | | | 1 | | | | 1 | | | | 1 | | | | 2 | | | | 1 | | | |
| 213 | | | 1 | | 1 | | 2 | | | | | | 2 | | | | 2 | | | | | | 1 | | | | 2 | | | | | | 1 | | | | 2 | | | | | | 2 | | | 2 | | | | | 1 | | | 2 | | | | | 1 | | | 2 | | | | | 1 | | | 2 | | | | | | 1 | | | 1 | | | | | | 1 | | | 2 | | | | | | 1 | | | 2 | | | | | | 1 | | | 2 | | | | | | 1 | | | 2 | | | | | | 2 | | | 2 | | | | | | 1 | | | 1 | | | | | | 1 | | | 2 | | | | | | 1 | | | 1 | | | | | | 1 | | | 1 | | | | | | 1 | | | 2 | | | | | 2 | | | 2 | | | | | 2 | | | 2 | | | | | 2 | | | 1 | | | | | 1 | | | 2 | | | | | 2 | | | 2 | | | | | 1 | | | | 2 | | | | 2 | | | | 2 | | | | 2 | | | | 2 | | | | 1 | | | | 2 | | | | 1 | | | | 2 | | | | 1 | | | |
| 214 | | | 2 | | 1 | | 2 | | | | | | 2 | | | | 2 | | | | | | 1 | | | | 2 | | | | | | 1 | | | | 2 | | | | | | 1 | | | 2 | | | | | 2 | | | 2 | | | | | 1 | | | 1 | | | | | 1 | | | 2 | | | | | | 1 | | | 1 | | | | | | 1 | | | 2 | | | | | | 2 | | | 2 | | | | | | 1 | | | 2 | | | | | | 1 | | | 1 | | | | | | 1 | | | 2 | | | | | | 1 | | | 2 | | | | | | 2 | | | 2 | | | | | | 1 | | | 2 | | | | | | 2 | | | 2 | | | | | | 1 | | | 2 | | | | | 1 | | | 2 | | | | | 2 | | | 2 | | | | | 1 | | | 1 | | | | | 1 | | | 2 | | | | | 2 | | | 2 | | | | | 1 | | | | 2 | | | | 1 | | | | 2 | | | | 1 | | | | 2 | | | | 1 | | | | 2 | | | | 1 | | | | 1 | | | | 1 | | | |
| Sample ID | HLD  *Supplementary Table (Continued)*  6 | | | | | | HLD  39 | | | | | | | | | | | | | | HLD  40 | | | | | | | | | | HLD  45 | | | | | | | | | | HLD  48 | | | | | | | | HLD  56 | | | | | | | | HLD  58 | | | | | | | | HLD  64 | | | | | | | | HLD  67 | | | | | | | | | HLD  70 | | | | | | | | | HLD  77 | | | | | | | | | HLD  81 | | | | | | | | | HLD  83 | | | | | | | | | HLD  84 | | | | | | | | | HLD  88 | | | | | | | | | HLD  92 | | | | | | | | | HLD  93 | | | | | | | | | HLD  97 | | | | | | | | | HLD  99 | | | | | | | | | HLD  101 | | | | | | | | HLD  111 | | | | | | | | HLD  114 | | | | | | | | HLD  118 | | | | | | HLD  122 | | | | | | | | HLD  124 | | | | | | | HLD  125 | | | | | | | | HLD  128 | | | | | | | | HLD  131 | | | | | | | | HLD  133 | | | | | | | | HLD  136 | | | | | | | |  |
| 215 | | | 2 | | 1 | | | | | | | 2 | | | | 2 | | | | 2 | | | | | | 2 | | | | 2 | | | | | | 1 | | | | 1 | | | | | 1 | | | 2 | | | | | 1 | | | 2 | | | | | 2 | | | 2 | | | | | 1 | | | 2 | | | | | | 1 | | | 2 | | | | | | 2 | | | 2 | | | | | | 1 | | | 1 | | | | | | 1 | | | 2 | | | | | | 2 | | | 2 | | | | | | 1 | | | 1 | | | | | | 1 | | | 1 | | | | | | 1 | | | 2 | | | | | | 2 | | | 2 | | | | | | 1 | | | 1 | | | | | | 1 | | | 2 | | | | | 1 | | | 2 | | | | | 2 | | | 2 | | | | | 1 | | | 1 | | | | | 1 | | | | 2 | | | | 2 | | | | 2 | | | | 2 | | | | 1 | | | | 1 | | | | 2 | | | | 1 | | | | 2 | | | | 2 | | | | 1 | | | | 1 | | | | 1 | | | | 1 | |
| 216 | | | 2 | | 1 | | | | | | | 2 | | | | 1 | | | | 1 | | | | | | 1 | | | | 2 | | | | | | 2 | | | | 2 | | | | | 1 | | | 1 | | | | | 1 | | | 2 | | | | | 1 | | | 1 | | | | | 1 | | | 2 | | | | | | 1 | | | 2 | | | | | | 1 | | | 2 | | | | | | 1 | | | 1 | | | | | | 1 | | | 2 | | | | | | 1 | | | 1 | | | | | | 1 | | | 2 | | | | | | 2 | | | 2 | | | | | | 1 | | | 1 | | | | | | 1 | | | 2 | | | | | | 1 | | | 1 | | | | | | 1 | | | 2 | | | | | 2 | | | 2 | | | | | 2 | | | 2 | | | | | 2 | | | 1 | | | | | 1 | | | | 2 | | | | 1 | | | | 2 | | | | 1 | | | | 2 | | | | 2 | | | | 2 | | | | 2 | | | | 2 | | | | 2 | | | | 2 | | | | 2 | | | | 2 | | | | 1 | |
| 217 | | | 2 | | 1 | | | | | | | 2 | | | | 2 | | | | 2 | | | | | | 2 | | | | 1 | | | | | | 1 | | | | 2 | | | | | 1 | | | 2 | | | | | 1 | | | 2 | | | | | 2 | | | 1 | | | | | 1 | | | 2 | | | | | | 1 | | | 1 | | | | | | 1 | | | 1 | | | | | | 1 | | | 2 | | | | | | 1 | | | 2 | | | | | | 1 | | | 1 | | | | | | 1 | | | 2 | | | | | | 1 | | | 1 | | | | | | 1 | | | 2 | | | | | | 1 | | | 2 | | | | | | 1 | | | 2 | | | | | | 2 | | | 2 | | | | | 1 | | | 2 | | | | | 2 | | | 2 | | | | | 1 | | | 2 | | | | | 2 | | | | 1 | | | | 1 | | | | 1 | | | | 1 | | | | 2 | | | | 1 | | | | 2 | | | | 2 | | | | 2 | | | | 2 | | | | 2 | | | | 2 | | | | 2 | | | | 1 | |
| 218 | | | 2 | | 2 | | | | | | | 2 | | | | 2 | | | | 1 | | | | | | 1 | | | | 2 | | | | | | 1 | | | | 2 | | | | | 1 | | | 2 | | | | | 1 | | | 2 | | | | | 1 | | | 1 | | | | | 1 | | | 1 | | | | | | 1 | | | 1 | | | | | | 1 | | | 2 | | | | | | 1 | | | 1 | | | | | | 1 | | | 1 | | | | | | 1 | | | 2 | | | | | | 1 | | | 2 | | | | | | 1 | | | 1 | | | | | | 1 | | | 1 | | | | | | 1 | | | 2 | | | | | | 1 | | | 1 | | | | | | 1 | | | 1 | | | | | 1 | | | 2 | | | | | 2 | | | 2 | | | | | 2 | | | 1 | | | | | 1 | | | | 2 | | | | 1 | | | | 1 | | | | 1 | | | | 1 | | | | 1 | | | | 2 | | | | 1 | | | | 2 | | | | 2 | | | | 2 | | | | 2 | | | | 1 | | | | 1 | |
| 219 | | | 2 | | 2 | | | | | | | 2 | | | | 1 | | | | 2 | | | | | | 2 | | | | 2 | | | | | | 1 | | | | 2 | | | | | 1 | | | 1 | | | | | 1 | | | 2 | | | | | 1 | | | 1 | | | | | 1 | | | 2 | | | | | | 1 | | | 2 | | | | | | 1 | | | 2 | | | | | | 2 | | | 2 | | | | | | 1 | | | 2 | | | | | | 1 | | | 2 | | | | | | 1 | | | 2 | | | | | | 1 | | | 2 | | | | | | 1 | | | 2 | | | | | | 2 | | | 2 | | | | | | 2 | | | 1 | | | | | | 1 | | | 2 | | | | | 2 | | | 2 | | | | | 2 | | | 2 | | | | | 1 | | | 1 | | | | | 1 | | | | 2 | | | | 2 | | | | 2 | | | | 1 | | | | 2 | | | | 2 | | | | 2 | | | | 2 | | | | 1 | | | | 1 | | | | 1 | | | | 1 | | | | 1 | | | | 1 | |
| 220 | | | 2 | | 1 | | | | | | | 2 | | | | 2 | | | | 1 | | | | | | 1 | | | | 2 | | | | | | 2 | | | | 1 | | | | | 1 | | | 2 | | | | | 2 | | | 2 | | | | | 1 | | | 1 | | | | | 1 | | | 1 | | | | | | 1 | | | 2 | | | | | | 2 | | | 2 | | | | | | 2 | | | 1 | | | | | | 1 | | | 2 | | | | | | 1 | | | 2 | | | | | | 2 | | | 2 | | | | | | 1 | | | 2 | | | | | | 1 | | | 2 | | | | | | 2 | | | 2 | | | | | | 1 | | | 1 | | | | | | 1 | | | 2 | | | | | 1 | | | 2 | | | | | 1 | | | 2 | | | | | 2 | | | 1 | | | | | 1 | | | | 2 | | | | 1 | | | | 2 | | | | 1 | | | | 2 | | | | 2 | | | | 2 | | | | 2 | | | | 1 | | | | 1 | | | | 2 | | | | 2 | | | | 2 | | | | 2 | |
| 221 | | | 2 | | 1 | | | | | | | 2 | | | | 1 | | | | 1 | | | | | | 1 | | | | 2 | | | | | | 1 | | | | 1 | | | | | 1 | | | 2 | | | | | 1 | | | 2 | | | | | 2 | | | 1 | | | | | 1 | | | 2 | | | | | | 1 | | | 2 | | | | | | 1 | | | 1 | | | | | | 1 | | | 1 | | | | | | 1 | | | 2 | | | | | | 2 | | | 1 | | | | | | 1 | | | 2 | | | | | | 2 | | | 2 | | | | | | 2 | | | 2 | | | | | | 2 | | | 2 | | | | | | 2 | | | 1 | | | | | | 1 | | | 2 | | | | | 2 | | | 2 | | | | | 2 | | | 2 | | | | | 1 | | | 1 | | | | | 1 | | | | 2 | | | | 1 | | | | 2 | | | | 1 | | | | 2 | | | | 2 | | | | 2 | | | | 2 | | | | 2 | | | | 2 | | | | 2 | | | | 2 | | | | 1 | | | | 1 | |
| 222 | | | 1 | | 1 | | | | | | | 2 | | | | 2 | | | | 2 | | | | | | 1 | | | | 2 | | | | | | 1 | | | | 2 | | | | | 1 | | | 2 | | | | | 2 | | | 2 | | | | | 1 | | | 1 | | | | | 1 | | | 1 | | | | | | 1 | | | 2 | | | | | | 2 | | | 2 | | | | | | 1 | | | 1 | | | | | | 1 | | | 2 | | | | | | 1 | | | 2 | | | | | | 2 | | | 2 | | | | | | 1 | | | 2 | | | | | | 2 | | | 2 | | | | | | 1 | | | 2 | | | | | | 2 | | | 1 | | | | | | 1 | | | 2 | | | | | 2 | | | 2 | | | | | 2 | | | 2 | | | | | 1 | | | 1 | | | | | 1 | | | | 2 | | | | 2 | | | | 2 | | | | 1 | | | | 1 | | | | 1 | | | | 1 | | | | 1 | | | | 2 | | | | 1 | | | | 1 | | | | 1 | | | | 1 | | | | 1 | |
| 223 | | | 2 | | 2 | | | | | | | 2 | | | | 2 | | | | 1 | | | | | | 1 | | | | 1 | | | | | | 1 | | | | 2 | | | | | 1 | | | 2 | | | | | 2 | | | 2 | | | | | 2 | | | 1 | | | | | 1 | | | 2 | | | | | | 1 | | | 1 | | | | | | 1 | | | 2 | | | | | | 1 | | | 1 | | | | | | 1 | | | 1 | | | | | | 2 | | | 1 | | | | | | 1 | | | 1 | | | | | | 1 | | | 2 | | | | | | 2 | | | 1 | | | | | | 1 | | | 2 | | | | | | 1 | | | 1 | | | | | | 1 | | | 2 | | | | | 1 | | | 2 | | | | | 2 | | | 2 | | | | | 2 | | | 2 | | | | | 1 | | | | 2 | | | | 1 | | | | 1 | | | | 1 | | | | 2 | | | | 1 | | | | 2 | | | | 2 | | | | 1 | | | | 1 | | | | 2 | | | | 1 | | | | 2 | | | | 2 | |
| 224 | | | 2 | | 1 | | | | | | | 2 | | | | 2 | | | | 1 | | | | | | 1 | | | | 2 | | | | | | 2 | | | | 2 | | | | | 2 | | | 2 | | | | | 2 | | | 2 | | | | | 2 | | | 1 | | | | | 1 | | | 2 | | | | | | 1 | | | 2 | | | | | | 1 | | | 1 | | | | | | 1 | | | 1 | | | | | | 1 | | | 2 | | | | | | 2 | | | 2 | | | | | | 2 | | | 2 | | | | | | 2 | | | 2 | | | | | | 1 | | | 2 | | | | | | 1 | | | 2 | | | | | | 2 | | | 1 | | | | | | 1 | | | 2 | | | | | 1 | | | 2 | | | | | 2 | | | 2 | | | | | 2 | | | 1 | | | | | 1 | | | | 1 | | | | 1 | | | | 2 | | | | 1 | | | | 2 | | | | 1 | | | | 2 | | | | 2 | | | | 2 | | | | 1 | | | | 2 | | | | 2 | | | | 2 | | | | 2 | |
| 225 | | | 2 | | 2 | | | | | | | 2 | | | | 2 | | | | 2 | | | | | | 2 | | | | 2 | | | | | | 1 | | | | 2 | | | | | 1 | | | 2 | | | | | 2 | | | 2 | | | | | 1 | | | 2 | | | | | 2 | | | 1 | | | | | | 1 | | | 1 | | | | | | 1 | | | 2 | | | | | | 1 | | | 2 | | | | | | 1 | | | 2 | | | | | | 2 | | | 1 | | | | | | 1 | | | 2 | | | | | | 1 | | | 2 | | | | | | 2 | | | 2 | | | | | | 1 | | | 1 | | | | | | 1 | | | 2 | | | | | | 1 | | | 1 | | | | | 1 | | | 2 | | | | | 1 | | | 2 | | | | | 1 | | | 1 | | | | | 1 | | | | 2 | | | | 2 | | | | 1 | | | | 1 | | | | 2 | | | | 2 | | | | 2 | | | | 2 | | | | 2 | | | | 1 | | | | 2 | | | | 2 | | | | 2 | | | | 1 | |
| 226 | | | 2 | | 1 | | | | | | | 2 | | | | 2 | | | | 2 | | | | | | 1 | | | | 2 | | | | | | 1 | | | | 2 | | | | | 2 | | | 2 | | | | | 1 | | | 1 | | | | | 1 | | | 1 | | | | | 1 | | | 1 | | | | | | 1 | | | 2 | | | | | | 1 | | | 1 | | | | | | 1 | | | 1 | | | | | | 1 | | | 2 | | | | | | 1 | | | 2 | | | | | | 1 | | | 1 | | | | | | 1 | | | 2 | | | | | | 1 | | | 2 | | | | | | 1 | | | 2 | | | | | | 2 | | | 1 | | | | | | 1 | | | 2 | | | | | 1 | | | 2 | | | | | 1 | | | 2 | | | | | 2 | | | 1 | | | | | 1 | | | | 2 | | | | 1 | | | | 2 | | | | 1 | | | | 2 | | | | 1 | | | | 2 | | | | 1 | | | | 2 | | | | 1 | | | | 2 | | | | 2 | | | | 1 | | | | 1 | |
| 227 | | | 1 | | 1 | | | | | | | 2 | | | | 2 | | | | 2 | | | | | | 1 | | | | 2 | | | | | | 2 | | | | 2 | | | | | 1 | | | 2 | | | | | 1 | | | 1 | | | | | 1 | | | 1 | | | | | 1 | | | 1 | | | | | | 1 | | | 2 | | | | | | 1 | | | 2 | | | | | | 1 | | | 1 | | | | | | 1 | | | 2 | | | | | | 1 | | | 2 | | | | | | 1 | | | 2 | | | | | | 2 | | | 2 | | | | | | 1 | | | 2 | | | | | | 1 | | | 2 | | | | | | 1 | | | 1 | | | | | | 1 | | | 2 | | | | | 2 | | | 2 | | | | | 2 | | | 2 | | | | | 2 | | | 1 | | | | | 1 | | | | 2 | | | | 2 | | | | 1 | | | | 1 | | | | 2 | | | | 1 | | | | 2 | | | | 1 | | | | 2 | | | | 1 | | | | 2 | | | | 1 | | | | 2 | | | | 2 | |
| 228 | | | 2 | | 1 | | | | | | | 2 | | | | 2 | | | | 2 | | | | | | 1 | | | | 2 | | | | | | 1 | | | | 2 | | | | | 2 | | | 1 | | | | | 1 | | | 1 | | | | | 1 | | | 1 | | | | | 1 | | | 2 | | | | | | 1 | | | 1 | | | | | | 1 | | | 2 | | | | | | 1 | | | 1 | | | | | | 1 | | | 2 | | | | | | 1 | | | 1 | | | | | | 1 | | | 2 | | | | | | 1 | | | 2 | | | | | | 1 | | | 2 | | | | | | 1 | | | 2 | | | | | | 2 | | | 1 | | | | | | 1 | | | 2 | | | | | 2 | | | 2 | | | | | 2 | | | 2 | | | | | 2 | | | 1 | | | | | 1 | | | | 2 | | | | 2 | | | | 1 | | | | 1 | | | | 2 | | | | 1 | | | | 1 | | | | 1 | | | | 2 | | | | 1 | | | | 2 | | | | 2 | | | | 2 | | | | 1 | |
| 229 | | | 2 | | 1 | | | | | | | 2 | | | | 2 | | | | 1 | | | | | | 1 | | | | 2 | | | | | | 1 | | | | 2 | | | | | 2 | | | 2 | | | | | 1 | | | 2 | | | | | 1 | | | 1 | | | | | 1 | | | 2 | | | | | | 2 | | | 2 | | | | | | 1 | | | 2 | | | | | | 1 | | | 1 | | | | | | 1 | | | 2 | | | | | | 2 | | | 2 | | | | | | 1 | | | 2 | | | | | | 1 | | | 1 | | | | | | 1 | | | 2 | | | | | | 1 | | | 1 | | | | | | 1 | | | 2 | | | | | | 1 | | | 1 | | | | | 1 | | | 2 | | | | | 2 | | | 2 | | | | | 1 | | | 1 | | | | | 1 | | | | 2 | | | | 1 | | | | 2 | | | | 2 | | | | 2 | | | | 1 | | | | 2 | | | | 2 | | | | 2 | | | | 2 | | | | 2 | | | | 2 | | | | 2 | | | | 1 | |
| 230 | | | 2 | | 1 | | | | | | | 2 | | | | 2 | | | | 2 | | | | | | 1 | | | | 1 | | | | | | 1 | | | | 2 | | | | | 2 | | | 1 | | | | | 1 | | | 2 | | | | | 2 | | | 2 | | | | | 1 | | | 2 | | | | | | 1 | | | 2 | | | | | | 1 | | | 2 | | | | | | 1 | | | 1 | | | | | | 1 | | | 1 | | | | | | 1 | | | 1 | | | | | | 1 | | | 2 | | | | | | 1 | | | 2 | | | | | | 2 | | | 1 | | | | | | 1 | | | 2 | | | | | | 1 | | | 1 | | | | | | 1 | | | 2 | | | | | 2 | | | 2 | | | | | 2 | | | 2 | | | | | 2 | | | 1 | | | | | 1 | | | | 2 | | | | 2 | | | | 1 | | | | 1 | | | | 2 | | | | 2 | | | | 2 | | | | 1 | | | | 2 | | | | 2 | | | | 2 | | | | 1 | | | | 2 | | | | 1 | |
| 231 | | | 2 | | 2 | | | | | | | 2 | | | | 1 | | | | 1 | | | | | | 1 | | | | 2 | | | | | | 1 | | | | 1 | | | | | 1 | | | 1 | | | | | 1 | | | 1 | | | | | 1 | | | 1 | | | | | 1 | | | 1 | | | | | | 1 | | | 2 | | | | | | 1 | | | 2 | | | | | | 1 | | | 1 | | | | | | 1 | | | 2 | | | | | | 1 | | | 1 | | | | | | 1 | | | 1 | | | | | | 1 | | | 2 | | | | | | 1 | | | 1 | | | | | | 1 | | | 2 | | | | | | 1 | | | 1 | | | | | | 1 | | | 2 | | | | | 1 | | | 2 | | | | | 2 | | | 2 | | | | | 1 | | | 1 | | | | | 1 | | | | 2 | | | | 2 | | | | 2 | | | | 1 | | | | 2 | | | | 1 | | | | 2 | | | | 2 | | | | 2 | | | | 1 | | | | 2 | | | | 1 | | | | 2 | | | | 1 | |
| 232 | | | 2 | | 2 | | | | | | | 2 | | | | 2 | | | | 1 | | | | | | 1 | | | | 1 | | | | | | 1 | | | | 1 | | | | | 1 | | | 1 | | | | | 1 | | | 2 | | | | | 2 | | | 1 | | | | | 1 | | | 2 | | | | | | 1 | | | 2 | | | | | | 1 | | | 2 | | | | | | 1 | | | 1 | | | | | | 1 | | | 2 | | | | | | 1 | | | 2 | | | | | | 2 | | | 2 | | | | | | 2 | | | 2 | | | | | | 1 | | | 1 | | | | | | 1 | | | 2 | | | | | | 2 | | | 1 | | | | | | 1 | | | 2 | | | | | 1 | | | 2 | | | | | 1 | | | 2 | | | | | 2 | | | 1 | | | | | 1 | | | | 2 | | | | 1 | | | | 2 | | | | 1 | | | | 2 | | | | 1 | | | | 2 | | | | 2 | | | | 2 | | | | 1 | | | | 2 | | | | 1 | | | | 2 | | | | 2 | |
| 233 | | | 2 | | 2 | | | | | | | 2 | | | | 1 | | | | 2 | | | | | | 1 | | | | 1 | | | | | | 1 | | | | 2 | | | | | 2 | | | 2 | | | | | 1 | | | 2 | | | | | 2 | | | 1 | | | | | 1 | | | 1 | | | | | | 1 | | | 2 | | | | | | 1 | | | 1 | | | | | | 1 | | | 2 | | | | | | 1 | | | 2 | | | | | | 1 | | | 2 | | | | | | 1 | | | 2 | | | | | | 2 | | | 2 | | | | | | 2 | | | 2 | | | | | | 1 | | | 2 | | | | | | 1 | | | 2 | | | | | | 1 | | | 2 | | | | | 1 | | | 2 | | | | | 2 | | | 2 | | | | | 2 | | | 2 | | | | | 1 | | | | 2 | | | | 2 | | | | 1 | | | | 1 | | | | 2 | | | | 2 | | | | 2 | | | | 2 | | | | 2 | | | | 2 | | | | 2 | | | | 2 | | | | 2 | | | | 1 | |
| 234 | | | 2 | | 1 | | | | | | | 2 | | | | 2 | | | | 2 | | | | | | 1 | | | | 2 | | | | | | 2 | | | | 2 | | | | | 1 | | | 1 | | | | | 1 | | | 2 | | | | | 2 | | | 1 | | | | | 1 | | | 1 | | | | | | 1 | | | 2 | | | | | | 1 | | | 2 | | | | | | 1 | | | 1 | | | | | | 1 | | | 2 | | | | | | 1 | | | 2 | | | | | | 1 | | | 2 | | | | | | 1 | | | 2 | | | | | | 1 | | | 2 | | | | | | 2 | | | 2 | | | | | | 2 | | | 1 | | | | | | 1 | | | 1 | | | | | 1 | | | 2 | | | | | 2 | | | 2 | | | | | 2 | | | 1 | | | | | 1 | | | | 1 | | | | 1 | | | | 2 | | | | 2 | | | | 2 | | | | 1 | | | | 2 | | | | 2 | | | | 2 | | | | 2 | | | | 2 | | | | 1 | | | | 2 | | | | 2 | |
| 235 | | | 2 | | 1 | | | | | | | 2 | | | | 2 | | | | 2 | | | | | | 1 | | | | 1 | | | | | | 1 | | | | 2 | | | | | 2 | | | 2 | | | | | 2 | | | 2 | | | | | 1 | | | 1 | | | | | 1 | | | 1 | | | | | | 1 | | | 1 | | | | | | 1 | | | 1 | | | | | | 1 | | | 1 | | | | | | 1 | | | 2 | | | | | | 2 | | | 1 | | | | | | 1 | | | 2 | | | | | | 1 | | | 2 | | | | | | 2 | | | 2 | | | | | | 1 | | | 2 | | | | | | 1 | | | 1 | | | | | | 1 | | | 1 | | | | | 1 | | | 2 | | | | | 1 | | | 2 | | | | | 1 | | | 1 | | | | | 1 | | | | 2 | | | | 2 | | | | 1 | | | | 1 | | | | 2 | | | | 1 | | | | 2 | | | | 2 | | | | 2 | | | | 2 | | | | 2 | | | | 1 | | | | 2 | | | | 1 | |
| 236 | | | 2 | | 2 | | | | | | | 2 | | | | 1 | | | | 2 | | | | | | 2 | | | | 1 | | | | | | 1 | | | | 2 | | | | | 2 | | | 1 | | | | | 1 | | | 2 | | | | | 1 | | | 1 | | | | | 1 | | | 2 | | | | | | 1 | | | 2 | | | | | | 2 | | | 1 | | | | | | 1 | | | 1 | | | | | | 1 | | | 2 | | | | | | 2 | | | 1 | | | | | | 1 | | | 2 | | | | | | 1 | | | 2 | | | | | | 1 | | | 2 | | | | | | 2 | | | 2 | | | | | | 2 | | | 1 | | | | | | 1 | | | 2 | | | | | 1 | | | 2 | | | | | 2 | | | 2 | | | | | 2 | | | 2 | | | | | 1 | | | | 2 | | | | 1 | | | | 1 | | | | 1 | | | | 2 | | | | 1 | | | | 2 | | | | 2 | | | | 2 | | | | 1 | | | | 2 | | | | 1 | | | | 2 | | | | 2 | |
| Sample ID  *Supplementary Table (Continued)* | HLD  6 | | | | | | HLD  39 | | | | | | | | | | | | | | HLD  40 | | | | | | | | | | HLD  45 | | | | | | | | | | HLD  48 | | | | | | | | HLD  56 | | | | | | | | HLD  58 | | | | | | | | HLD  64 | | | | | | | | HLD  67 | | | | | | | | | HLD  70 | | | | | | | | | HLD  77 | | | | | | | | | HLD  81 | | | | | | | | | HLD  83 | | | | | | | | | HLD  84 | | | | | | | | | HLD  88 | | | | | | | | | HLD  92 | | | | | | | | | HLD  93 | | | | | | | | | HLD  97 | | | | | | | | | HLD  99 | | | | | | | | | HLD  101 | | | | | | | | HLD  111 | | | | | | | | HLD  114 | | | | | | | | HLD  118 | | | | | | HLD  122 | | | | | | | | HLD  124 | | | | | | | HLD  125 | | | | | | | | HLD  128 | | | | | | | | HLD  131 | | | | | | | | HLD  133 | | | | | | | | HLD  136 | | | | | | | |  |
| 237 | | | 2 | | 2 | | | | | | 2 | | | | 1 | | | | 1 | | | | | | 1 | | | | 1 | | | | | | 1 | | | | 2 | | | | | 2 | | | 1 | | | | | 1 | | | 2 | | | | | 2 | | | 1 | | | | | 1 | | | 2 | | | | | | 2 | | | 2 | | | | | | 1 | | | 1 | | | | | | 1 | | | 1 | | | | | | 1 | | | 2 | | | | | | 2 | | | 1 | | | | | | 1 | | | 2 | | | | | | 2 | | | 2 | | | | | | 1 | | | 2 | | | | | | 1 | | | 2 | | | | | | 1 | | | 1 | | | | | | 1 | | | 2 | | | | | 2 | | | 2 | | | | | 2 | | | 2 | | | | | 2 | | | 1 | | | | | 1 | | | | 2 | | | | 2 | | | | 2 | | | | 1 | | | | 2 | | | | 1 | | | | 1 | | | | 1 | | | | 1 | | | | 1 | | | | 2 | | | | 1 | | | | 2 | | | | 2 | | |
| 238 | | | 2 | | 2 | | | | | | 2 | | | | 1 | | | | 1 | | | | | | 1 | | | | 1 | | | | | | 1 | | | | 2 | | | | | 1 | | | 2 | | | | | 2 | | | 2 | | | | | 1 | | | 2 | | | | | 1 | | | 2 | | | | | | 2 | | | 1 | | | | | | 1 | | | 2 | | | | | | 2 | | | 1 | | | | | | 1 | | | 2 | | | | | | 2 | | | 2 | | | | | | 2 | | | 2 | | | | | | 1 | | | 2 | | | | | | 2 | | | 2 | | | | | | 1 | | | 2 | | | | | | 1 | | | 2 | | | | | | 1 | | | 2 | | | | | 1 | | | 2 | | | | | 2 | | | 2 | | | | | 2 | | | 2 | | | | | 1 | | | | 2 | | | | 2 | | | | 1 | | | | 1 | | | | 1 | | | | 1 | | | | 2 | | | | 1 | | | | 2 | | | | 2 | | | | 2 | | | | 1 | | | | 2 | | | | 1 | | |
| 239 | | | 2 | | 1 | | | | | | 2 | | | | 2 | | | | 2 | | | | | | 2 | | | | 1 | | | | | | 1 | | | | 2 | | | | | 1 | | | 2 | | | | | 1 | | | 2 | | | | | 2 | | | 1 | | | | | 1 | | | 2 | | | | | | 1 | | | 1 | | | | | | 1 | | | 1 | | | | | | 1 | | | 2 | | | | | | 1 | | | 2 | | | | | | 1 | | | 1 | | | | | | 1 | | | 2 | | | | | | 1 | | | 1 | | | | | | 1 | | | 2 | | | | | | 1 | | | 2 | | | | | | 1 | | | 2 | | | | | | 2 | | | 2 | | | | | 1 | | | 2 | | | | | 2 | | | 2 | | | | | 1 | | | 2 | | | | | 2 | | | | 1 | | | | 1 | | | | 1 | | | | 1 | | | | 2 | | | | 1 | | | | 2 | | | | 2 | | | | 2 | | | | 2 | | | | 2 | | | | 2 | | | | 2 | | | | 1 | | |
| 240 | | | 2 | | 1 | | | | | | 2 | | | | 1 | | | | 1 | | | | | | 1 | | | | 2 | | | | | | 1 | | | | 1 | | | | | 1 | | | 2 | | | | | 1 | | | 2 | | | | | 1 | | | 1 | | | | | 1 | | | 2 | | | | | | 2 | | | 2 | | | | | | 1 | | | 2 | | | | | | 1 | | | 1 | | | | | | 1 | | | 2 | | | | | | 1 | | | 2 | | | | | | 1 | | | 2 | | | | | | 1 | | | 2 | | | | | | 2 | | | 2 | | | | | | 2 | | | 2 | | | | | | 1 | | | 1 | | | | | | 1 | | | 2 | | | | | 2 | | | 2 | | | | | 2 | | | 2 | | | | | 1 | | | 1 | | | | | 1 | | | | 2 | | | | 2 | | | | 2 | | | | 1 | | | | 2 | | | | 1 | | | | 2 | | | | 2 | | | | 2 | | | | 1 | | | | 2 | | | | 1 | | | | 2 | | | | 1 | | |
| 241 | | | 1 | | 1 | | | | | | 2 | | | | 2 | | | | 2 | | | | | | 1 | | | | 2 | | | | | | 1 | | | | 1 | | | | | 1 | | | 2 | | | | | 1 | | | 2 | | | | | 1 | | | 1 | | | | | 1 | | | 1 | | | | | | 1 | | | 1 | | | | | | 1 | | | 2 | | | | | | 1 | | | 2 | | | | | | 1 | | | 2 | | | | | | 1 | | | 1 | | | | | | 1 | | | 1 | | | | | | 1 | | | 2 | | | | | | 2 | | | 2 | | | | | | 2 | | | 1 | | | | | | 1 | | | 2 | | | | | | 1 | | | 2 | | | | | 1 | | | 2 | | | | | 2 | | | 2 | | | | | 2 | | | 1 | | | | | 1 | | | | 2 | | | | 2 | | | | 1 | | | | 1 | | | | 2 | | | | 2 | | | | 2 | | | | 1 | | | | 2 | | | | 1 | | | | 2 | | | | 1 | | | | 1 | | | | 1 | | |
| 242 | | | 2 | | 1 | | | | | | 2 | | | | 1 | | | | 1 | | | | | | 1 | | | | 1 | | | | | | 1 | | | | 2 | | | | | 1 | | | 1 | | | | | 1 | | | 2 | | | | | 2 | | | 1 | | | | | 1 | | | 2 | | | | | | 2 | | | 2 | | | | | | 1 | | | 2 | | | | | | 1 | | | 1 | | | | | | 1 | | | 1 | | | | | | 1 | | | 2 | | | | | | 1 | | | 2 | | | | | | 1 | | | 2 | | | | | | 2 | | | 1 | | | | | | 1 | | | 2 | | | | | | 1 | | | 1 | | | | | | 1 | | | 2 | | | | | 1 | | | 2 | | | | | 1 | | | 2 | | | | | 2 | | | 2 | | | | | 1 | | | | 2 | | | | 1 | | | | 1 | | | | 1 | | | | 2 | | | | 1 | | | | 2 | | | | 2 | | | | 2 | | | | 2 | | | | 2 | | | | 1 | | | | 2 | | | | 1 | | |
| 243 | | | 1 | | 1 | | | | | | 2 | | | | 2 | | | | 1 | | | | | | 1 | | | | 2 | | | | | | 1 | | | | 2 | | | | | 1 | | | 1 | | | | | 1 | | | 2 | | | | | 1 | | | 1 | | | | | 1 | | | 2 | | | | | | 1 | | | 1 | | | | | | 1 | | | 1 | | | | | | 1 | | | 1 | | | | | | 1 | | | 2 | | | | | | 2 | | | 1 | | | | | | 1 | | | 2 | | | | | | 2 | | | 1 | | | | | | 1 | | | 2 | | | | | | 2 | | | 2 | | | | | | 2 | | | 2 | | | | | | 2 | | | 1 | | | | | 1 | | | 2 | | | | | 2 | | | 2 | | | | | 1 | | | 1 | | | | | 1 | | | | 2 | | | | 2 | | | | 1 | | | | 1 | | | | 2 | | | | 1 | | | | 2 | | | | 2 | | | | 2 | | | | 2 | | | | 2 | | | | 2 | | | | 2 | | | | 1 | | |
| 244 | | | 1 | | 1 | | | | | | 2 | | | | 2 | | | | 2 | | | | | | 1 | | | | 1 | | | | | | 1 | | | | 2 | | | | | 2 | | | 1 | | | | | 1 | | | 2 | | | | | 1 | | | 2 | | | | | 1 | | | 1 | | | | | | 1 | | | 2 | | | | | | 1 | | | 1 | | | | | | 1 | | | 2 | | | | | | 1 | | | 2 | | | | | | 1 | | | 2 | | | | | | 2 | | | 2 | | | | | | 2 | | | 2 | | | | | | 1 | | | 2 | | | | | | 1 | | | 2 | | | | | | 2 | | | 2 | | | | | | 1 | | | 2 | | | | | 2 | | | 2 | | | | | 2 | | | 2 | | | | | 2 | | | 2 | | | | | 1 | | | | 2 | | | | 2 | | | | 2 | | | | 2 | | | | 2 | | | | 1 | | | | 2 | | | | 2 | | | | 2 | | | | 1 | | | | 2 | | | | 1 | | | | 2 | | | | 2 | | |
| 245 | | | 2 | | 1 | | | | | | 2 | | | | 2 | | | | 2 | | | | | | 1 | | | | 1 | | | | | | 1 | | | | 2 | | | | | 2 | | | 2 | | | | | 2 | | | 2 | | | | | 2 | | | 2 | | | | | 1 | | | 2 | | | | | | 2 | | | 1 | | | | | | 1 | | | 1 | | | | | | 1 | | | 2 | | | | | | 1 | | | 2 | | | | | | 1 | | | 1 | | | | | | 1 | | | 2 | | | | | | 2 | | | 2 | | | | | | 2 | | | 1 | | | | | | 1 | | | 2 | | | | | | 2 | | | 1 | | | | | | 1 | | | 2 | | | | | 1 | | | 2 | | | | | 2 | | | 2 | | | | | 2 | | | 1 | | | | | 1 | | | | 2 | | | | 2 | | | | 2 | | | | 1 | | | | 1 | | | | 1 | | | | 2 | | | | 1 | | | | 2 | | | | 1 | | | | 1 | | | | 1 | | | | 2 | | | | 2 | | |
| 246 | | | 1 | | 1 | | | | | | 2 | | | | 2 | | | | 2 | | | | | | 1 | | | | 2 | | | | | | 1 | | | | 1 | | | | | 1 | | | 1 | | | | | 1 | | | 2 | | | | | 2 | | | 1 | | | | | 1 | | | 1 | | | | | | 1 | | | 2 | | | | | | 1 | | | 2 | | | | | | 2 | | | 2 | | | | | | 1 | | | 2 | | | | | | 1 | | | 2 | | | | | | 1 | | | 2 | | | | | | 2 | | | 1 | | | | | | 1 | | | 2 | | | | | | 1 | | | 2 | | | | | | 1 | | | 1 | | | | | | 1 | | | 2 | | | | | 2 | | | 2 | | | | | 2 | | | 2 | | | | | 2 | | | 1 | | | | | 1 | | | | 2 | | | | 1 | | | | 1 | | | | 1 | | | | 2 | | | | 1 | | | | 2 | | | | 2 | | | | 2 | | | | 1 | | | | 2 | | | | 2 | | | | 2 | | | | 1 | | |
| 247 | | | 2 | | 2 | | | | | | 2 | | | | 1 | | | | 1 | | | | | | 1 | | | | 1 | | | | | | 1 | | | | 1 | | | | | 1 | | | 2 | | | | | 1 | | | 2 | | | | | 1 | | | 1 | | | | | 1 | | | 1 | | | | | | 1 | | | 2 | | | | | | 2 | | | 2 | | | | | | 2 | | | 2 | | | | | | 2 | | | 2 | | | | | | 2 | | | 1 | | | | | | 1 | | | 2 | | | | | | 1 | | | 2 | | | | | | 2 | | | 2 | | | | | | 2 | | | 2 | | | | | | 2 | | | 2 | | | | | | 1 | | | 2 | | | | | 1 | | | 2 | | | | | 2 | | | 2 | | | | | 1 | | | 1 | | | | | 1 | | | | 2 | | | | 2 | | | | 2 | | | | 1 | | | | 2 | | | | 2 | | | | 2 | | | | 1 | | | | 2 | | | | 2 | | | | 1 | | | | 1 | | | | 1 | | | | 1 | | |
| 248 | | | 2 | | 1 | | | | | | 2 | | | | 1 | | | | 2 | | | | | | 1 | | | | 2 | | | | | | 1 | | | | 2 | | | | | 1 | | | 1 | | | | | 1 | | | 2 | | | | | 1 | | | 2 | | | | | 1 | | | 2 | | | | | | 1 | | | 1 | | | | | | 1 | | | 2 | | | | | | 2 | | | 1 | | | | | | 1 | | | 2 | | | | | | 2 | | | 1 | | | | | | 1 | | | 1 | | | | | | 1 | | | 2 | | | | | | 1 | | | 2 | | | | | | 2 | | | 2 | | | | | | 1 | | | 1 | | | | | | 1 | | | 2 | | | | | 1 | | | 2 | | | | | 2 | | | 2 | | | | | 2 | | | 1 | | | | | 1 | | | | 2 | | | | 1 | | | | 2 | | | | 1 | | | | 2 | | | | 1 | | | | 2 | | | | 1 | | | | 2 | | | | 2 | | | | 2 | | | | 1 | | | | 2 | | | | 2 | | |
| 249 | | | 2 | | 2 | | | | | | 1 | | | | 1 | | | | 2 | | | | | | 2 | | | | 2 | | | | | | 2 | | | | 2 | | | | | 1 | | | 2 | | | | | 1 | | | 2 | | | | | 2 | | | 2 | | | | | 1 | | | 2 | | | | | | 1 | | | 2 | | | | | | 1 | | | 2 | | | | | | 1 | | | 2 | | | | | | 1 | | | 2 | | | | | | 1 | | | 2 | | | | | | 1 | | | 2 | | | | | | 1 | | | 2 | | | | | | 1 | | | 2 | | | | | | 2 | | | 2 | | | | | | 2 | | | 1 | | | | | | 1 | | | 2 | | | | | 2 | | | 2 | | | | | 1 | | | 2 | | | | | 2 | | | 1 | | | | | 1 | | | | 2 | | | | 2 | | | | 1 | | | | 1 | | | | 2 | | | | 1 | | | | 2 | | | | 2 | | | | 2 | | | | 1 | | | | 1 | | | | 1 | | | | 2 | | | | 2 | | |
| 250 | | | 2 | | 2 | | | | | | 2 | | | | 2 | | | | 1 | | | | | | 1 | | | | 1 | | | | | | 1 | | | | 2 | | | | | 1 | | | 2 | | | | | 1 | | | 2 | | | | | 1 | | | 1 | | | | | 1 | | | 1 | | | | | | 1 | | | 2 | | | | | | 1 | | | 2 | | | | | | 1 | | | 1 | | | | | | 1 | | | 2 | | | | | | 1 | | | 1 | | | | | | 1 | | | 2 | | | | | | 1 | | | 2 | | | | | | 1 | | | 1 | | | | | | 1 | | | 2 | | | | | | 2 | | | 2 | | | | | | 1 | | | 2 | | | | | 2 | | | 1 | | | | | 1 | | | 2 | | | | | 1 | | | 1 | | | | | 1 | | | | 2 | | | | 1 | | | | 2 | | | | 2 | | | | 2 | | | | 1 | | | | 2 | | | | 1 | | | | 2 | | | | 1 | | | | 2 | | | | 2 | | | | 2 | | | | 2 | | |
| 251 | | | 1 | | 1 | | | | | | 2 | | | | 1 | | | | 2 | | | | | | 1 | | | | 2 | | | | | | 1 | | | | 2 | | | | | 2 | | | 1 | | | | | 1 | | | 2 | | | | | 1 | | | 1 | | | | | 1 | | | 2 | | | | | | 1 | | | 1 | | | | | | 1 | | | 2 | | | | | | 1 | | | 2 | | | | | | 1 | | | 2 | | | | | | 2 | | | 1 | | | | | | 1 | | | 2 | | | | | | 1 | | | 2 | | | | | | 1 | | | 2 | | | | | | 2 | | | 2 | | | | | | 2 | | | 2 | | | | | | 1 | | | 1 | | | | | 1 | | | 2 | | | | | 2 | | | 2 | | | | | 1 | | | 1 | | | | | 1 | | | | 2 | | | | 2 | | | | 2 | | | | 1 | | | | 2 | | | | 1 | | | | 2 | | | | 2 | | | | 2 | | | | 1 | | | | 2 | | | | 2 | | | | 2 | | | | 2 | | |
| 252 | | | 2 | | 1 | | | | | | 2 | | | | 2 | | | | 2 | | | | | | 1 | | | | 2 | | | | | | 1 | | | | 1 | | | | | 1 | | | 2 | | | | | 2 | | | 2 | | | | | 1 | | | 1 | | | | | 1 | | | 1 | | | | | | 1 | | | 1 | | | | | | 1 | | | 2 | | | | | | 2 | | | 1 | | | | | | 1 | | | 1 | | | | | | 1 | | | 1 | | | | | | 1 | | | 2 | | | | | | 1 | | | 2 | | | | | | 1 | | | 2 | | | | | | 1 | | | 2 | | | | | | 2 | | | 2 | | | | | | 1 | | | 2 | | | | | 1 | | | 2 | | | | | 2 | | | 2 | | | | | 1 | | | 1 | | | | | 1 | | | | 2 | | | | 1 | | | | 2 | | | | 2 | | | | 2 | | | | 1 | | | | 2 | | | | 1 | | | | 2 | | | | 1 | | | | 2 | | | | 1 | | | | 1 | | | | 1 | | |

1 represents insertion; 2 represents deletion
