# Supplementary material for: Genetic variation and forensic efficiency of autosomal insertion/deletion polymorphisms in Chinese Bai ethnic group: phylogenetic analysis to other populations
Source: Oncotarget. 2017 Apr 17;8(24):39582–91. doi: 10.18632/oncotarget.17137 (PMC5503634; doi:10.18632/oncotarget.17137)
Supplement: Supplementary file 3 [file oncotarget-08-39582-s003.doc]

| Supplementary Table 2. The values of *D_A_* of pairwise populations among Chinese Bai group and referenced populations. | | | | | | | | | | | | | | | | | | |  |  |  |  |  |  |  |
| --- | --- | --- | --- | --- | --- | --- | --- | --- | --- | --- | --- | --- | --- | --- | --- | --- | --- | --- | --- | --- | --- | --- | --- | --- | --- |
| Populations | Uruguyan | Dane | Basque | Central Spanish | Hungarian | Uyghur | Uyghur1 | Uyghur2 | Kazak | Chengdu Han | Zhuang | Dong | Miao | Henan Han | Beijing Han | Beijing Han1 | Tibetan1 | Tibetan2 | Tibetan | Yi | Guangdong Han | Shanghai Han | Xibe | South Korean | She |
| Dane | 0.0039 |  |  |  |  |  |  |  |  |  |  |  |  |  |  |  |  |  |  |  |  |  |  |  |  |
| Basque | 0.0043 | 0.0048 |  |  |  |  |  |  |  |  |  |  |  |  |  |  |  |  |  |  |  |  |  |  |  |
| Central Spanish | 0.0023 | 0.0030 | 0.0033 |  |  |  |  |  |  |  |  |  |  |  |  |  |  |  |  |  |  |  |  |  |  |
| Hungarian | 0.0021 | 0.0026 | 0.0045 | 0.0022 |  |  |  |  |  |  |  |  |  |  |  |  |  |  |  |  |  |  |  |  |  |
| Uyghur | 0.0057 | 0.0083 | 0.0096 | 0.0069 | 0.0068 |  |  |  |  |  |  |  |  |  |  |  |  |  |  |  |  |  |  |  |  |
| Uyghur1 | 0.0069 | 0.0101 | 0.0115 | 0.0088 | 0.0083 | 0.0029 |  |  |  |  |  |  |  |  |  |  |  |  |  |  |  |  |  |  |  |
| Uyghur2 | 0.0053 | 0.0083 | 0.0093 | 0.0073 | 0.0069 | 0.0022 | 0.0012 |  |  |  |  |  |  |  |  |  |  |  |  |  |  |  |  |  |  |
| Kazak | 0.0067 | 0.0093 | 0.0111 | 0.0085 | 0.0084 | 0.0013 | 0.0030 | 0.0027 |  |  |  |  |  |  |  |  |  |  |  |  |  |  |  |  |  |
| Chengdu Han | 0.0235 | 0.0256 | 0.0256 | 0.0245 | 0.0252 | 0.0143 | 0.0076 | 0.0108 | 0.0136 |  |  |  |  |  |  |  |  |  |  |  |  |  |  |  |  |
| Zhuang | 0.0237 | 0.0263 | 0.0254 | 0.0242 | 0.0262 | 0.0159 | 0.0090 | 0.0121 | 0.0148 | 0.0029 |  |  |  |  |  |  |  |  |  |  |  |  |  |  |  |
| Dong | 0.0341 | 0.0355 | 0.0357 | 0.0355 | 0.0358 | 0.0239 | 0.0156 | 0.0197 | 0.0213 | 0.0044 | 0.0042 |  |  |  |  |  |  |  |  |  |  |  |  |  |  |
| Miao | 0.0287 | 0.0299 | 0.0322 | 0.0303 | 0.0298 | 0.0177 | 0.0114 | 0.0154 | 0.0165 | 0.0045 | 0.0040 | 0.0039 |  |  |  |  |  |  |  |  |  |  |  |  |  |
| Henan Han | 0.0240 | 0.0271 | 0.0268 | 0.0258 | 0.0261 | 0.0144 | 0.0077 | 0.0109 | 0.0136 | 0.0011 | 0.0035 | 0.0045 | 0.0052 |  |  |  |  |  |  |  |  |  |  |  |  |
| Beijing Han | 0.0230 | 0.0251 | 0.0270 | 0.0262 | 0.0255 | 0.0100 | 0.0108 | 0.0121 | 0.0083 | 0.0115 | 0.0140 | 0.0169 | 0.0133 | 0.0111 |  |  |  |  |  |  |  |  |  |  |  |
| Beijing Han1 | 0.0237 | 0.0262 | 0.0262 | 0.0254 | 0.0258 | 0.0138 | 0.0078 | 0.0104 | 0.0127 | 0.0014 | 0.0046 | 0.0054 | 0.0067 | 0.0006 | 0.0109 |  |  |  |  |  |  |  |  |  |  |
| Tibetan1 | 0.0219 | 0.0248 | 0.0258 | 0.0245 | 0.0239 | 0.0112 | 0.0066 | 0.0086 | 0.0102 | 0.0033 | 0.0051 | 0.0063 | 0.0052 | 0.0021 | 0.0086 | 0.0018 |  |  |  |  |  |  |  |  |  |
| Tibetan2 | 0.0208 | 0.0243 | 0.0264 | 0.0234 | 0.0230 | 0.0107 | 0.0056 | 0.0076 | 0.0092 | 0.0037 | 0.0066 | 0.0088 | 0.0079 | 0.0027 | 0.0079 | 0.0023 | 0.0014 |  |  |  |  |  |  |  |  |
| Tibetan | 0.0199 | 0.0226 | 0.0258 | 0.0231 | 0.0222 | 0.0093 | 0.0075 | 0.0089 | 0.0074 | 0.0094 | 0.0124 | 0.0152 | 0.0119 | 0.0090 | 0.0029 | 0.0088 | 0.0057 | 0.0039 |  |  |  |  |  |  |  |
| Yi | 0.0250 | 0.0270 | 0.0285 | 0.0263 | 0.0271 | 0.0151 | 0.0097 | 0.0126 | 0.0138 | 0.0026 | 0.0051 | 0.0064 | 0.0056 | 0.0020 | 0.0105 | 0.0023 | 0.0031 | 0.0036 | 0.0081 |  |  |  |  |  |  |
| Guangdong Han | 0.0244 | 0.0265 | 0.0268 | 0.0269 | 0.0275 | 0.0118 | 0.0120 | 0.0136 | 0.0100 | 0.0102 | 0.0117 | 0.0146 | 0.0116 | 0.0109 | 0.0019 | 0.0112 | 0.0097 | 0.0103 | 0.0055 | 0.0109 |  |  |  |  |  |
| Shanghai Han | 0.0240 | 0.0264 | 0.0270 | 0.0268 | 0.0271 | 0.0114 | 0.0114 | 0.0129 | 0.0096 | 0.0101 | 0.0128 | 0.0159 | 0.0132 | 0.0103 | 0.0011 | 0.0103 | 0.0088 | 0.0087 | 0.0038 | 0.0101 | 0.0006 |  |  |  |  |
| Xibe | 0.0203 | 0.0227 | 0.0236 | 0.0226 | 0.0231 | 0.0092 | 0.0090 | 0.0101 | 0.0068 | 0.0086 | 0.0112 | 0.0144 | 0.0127 | 0.0086 | 0.0022 | 0.0079 | 0.0073 | 0.0065 | 0.0037 | 0.0078 | 0.0023 | 0.0015 |  |  |  |
| South Korean | 0.0258 | 0.0288 | 0.0287 | 0.0288 | 0.0295 | 0.0135 | 0.0120 | 0.0137 | 0.0115 | 0.0084 | 0.0120 | 0.0139 | 0.0126 | 0.0085 | 0.0024 | 0.0082 | 0.0077 | 0.0072 | 0.0038 | 0.0080 | 0.0017 | 0.0008 | 0.0016 |  |  |
| She | 0.0255 | 0.0275 | 0.0288 | 0.0285 | 0.0289 | 0.0133 | 0.0138 | 0.0149 | 0.0112 | 0.0124 | 0.0136 | 0.0162 | 0.0124 | 0.0127 | 0.0023 | 0.0128 | 0.0108 | 0.0113 | 0.0065 | 0.0120 | 0.0015 | 0.0019 | 0.0032 | 0.0028 |  |
| Bai | 0.0213 | 0.0236 | 0.0238 | 0.0232 | 0.0229 | 0.0111 | 0.0063 | 0.0085 | 0.0107 | 0.0019 | 0.0038 | 0.0052 | 0.0041 | 0.0014 | 0.0090 | 0.0017 | 0.0012 | 0.0023 | 0.0066 | 0.0023 | 0.0089 | 0.0087 | 0.0073 | 0.0072 | 0.0108 |
